# Vegetal residue‐based formulation of *Trichoderma ossianense*, a new indigenous vineyard species adapted to alkaline pH with potential biocontrol ability against Black‐foot disease pathogens

**DOI:** 10.1002/ps.70417

**Published:** 2025-12-06

**Authors:** Laura Zanfaño, Guzmán Carro‐Huerga, Álvaro Rodríguez‐González, Sara Mayo‐Prieto, Daniela Ramírez‐Lozano, Rosa E Cardoza, Santiago Gutiérrez, Pedro A Casquero

**Affiliations:** ^1^ Grupo Universitario de Investigación en Ingeniería y Agricultura Sostenible, Instituto de Medio Ambiente, Recursos Naturales y Biodiversidad, Departamento de Ingeniería y Ciencias Agrarias Universidad de León León Spain; ^2^ Área de Microbiología, Grupo Universitario de Investigación en Ingeniería y Agricultura Sostenible, Instituto de Medio Ambiente, Recursos Naturales y Biodiversidad Universidad de León León Spain

**Keywords:** indigenous, alkaline soil, *Ilyonectria*, phylogenetic, vegetal residues, bioeconomy

## Abstract

**BACKGROUND:**

Fungi of the *Trichoderma* genus are used in vineyards as biological control agents mainly against grapevine trunk diseases. The use of indigenous strains of this fungal genus favors their efficacy since they are optimally adapted to the environmental conditions. Some factors, such as the pH of soils colonized by *Trichoderma*, are essential to guarantee its efficacy against grapevine pathogens. For this reason, the aim of this study was to search for *Trichoderma* strains adapted to soils with alkaline pH, predominant in different wine‐growing areas of Spain, able to combat the pathogen *Ilyonectria sp.*, the causal agent of the grapevine black‐foot disease.

**RESULTS:**

This study identified a new *Trichoderma* species, *T. ossianense*, isolated from grapevine roots. This isolate is able to grow in alkaline pH and shows efficacy in the biocontrol of pathogens of the *Ilyonectria* and *Dactylonectria* genera, which cause black‐foot disease in grapevine. Algae residue bioformulations of *T. ossianense* maintain spore viability at low temperatures during storage periods, whereas the wheat residue‐based formulation shows higher ability to supply nutrients and promote *Trichoderma* development under field conditions.

**CONCLUSION:**

This study opens up a wide range of possibilities for the protection of vineyards in areas with alkaline pH, as well as for the prevention of *Ilyonectria* sp. and *Dactylonectria* sp. in nurseries and young vineyards using vegetal residue‐based formulations of *T. ossianense*. These results highlight *T. ossianense* as a reliable, safe, and promising biocontrol agent against grapevine Black‐foot disease. © 2025 The Author(s). *Pest Management Science* published by John Wiley & Sons Ltd on behalf of Society of Chemical Industry.

## Graphical abstract

The study identifies *Trichoderma ossianense*, was can be isolated from grapevine roots, is capable of growing at alkaline pH and controls black‐foot pathogens, highlighting the vegetal residue‐based formulations that preserve its viability.

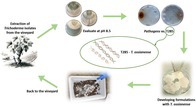

## 1 INTRODUCTION

*Trichoderma* (Hypocreaceae, Hypocreales, Sordariomycetes, and Ascomycota) is one of the most important fungal genera currently available as a biological control agent (BCA).^1^ The phylogeny of the genus *Trichoderma* is a subject of great complexity due to the great genetic diversity and plasticity of its species. At present, thanks to advances in molecular techniques, the number of identified species has increased significantly, with more than 450 species being recognized.^2^ Phylogenetic analyses have identified several important clades and sections within the genus. According to these studies, most species of *Trichoderma* could be clustered into three big groups, clade *Harzianum/Virens*, section *Trichoderma*, and section *Longibrachiatum*.^3^ Nowadays, more accurate and advisable procedures to identify new *Trichoderma* species are being used, such as next‐generation sequencing techniques and the standardized protocol of the International Commission on *Trichoderma* Taxonomy (ICTT, https://trichoderma.info/2021/04/26/molecular-identification-protocol-for-trichoderma/).

The genus *Trichoderma* is characterized by the variety of mechanisms that it employs as BCA, such as mycoparasitism, antibiosis, competition with pathogens, production of extracellular hydrolytic enzymes, and the induction of plant defense response.^4^ Currently, the use of indigenous *Trichoderma* strains is of great interest in viticulture, especially for the control of grapevine trunk diseases (GTDs).^5^, ^6^, ^7^, ^8^, ^9^ GTDs are an important problem worldwide in viticulture and there are no curative methods, only preventative ones.^10^ It is therefore of great relevance to find *Trichoderma* strains adapted to different environmental conditions such as temperature or humidity and other soil conditions, such as pH.^11^, ^12^ Among GTDs, black‐foot disease stands as one of the most destructive grapevine trunk diseases affecting vineyards worldwide, particularly damaging nursery plants and young vineyards. This disease has gained increasing attention over recent decades due to its economic impact on global viticulture. Black‐foot disease is currently considered as one of the most significant threats facing young grapevine establishment.^13^

The taxonomy of fungi causing black‐foot disease has undergone significant revisions in recent years. Initially attributed primarily to species in the genus *Cylindrocarpon*, recent taxonomic studies based on morphological features and phylogenetic analyses have revealed a complex of multiple genera responsible for the disease. Thus, modern classification divides the causal agents of black‐foot disease into several genera: *Campylocarpon*, *Cylindrocladiella*, *Dactylonectria*, *Ilyonectria*, *Neonectria*, and *Thelonectria*. The *Dactylonectria* genus includes species such as *D. torresensis* (the most prevalent species in Spain, Portugal, and Italy), *D. macrodidyma*, *D. alcacerensis*, *D. novozelandica*, *D. pauciseptata*, and the newly described *D. riojan*. Key species of *Ilyonectria* include *I. liriodendri*, *I. robusta*, *I. pseudodestructans*, and the recently described *I. vivaria*.^14^ The symptoms produced by these fungi are dark, sunken, and necrotic lesions on roots, and black discoloration and necrosis of the woody tissue from the base of the rootstock. Other symptoms, such as reduced vigor, sparse foliage, shortened internodes, and small leaves with chlorosis and interveinal necrosis, may also be observed, leading to the death of the plant.^15^, ^16^

Recent studies have demonstrated the efficacy of *Trichoderma* in the control of several black‐foot pathogens. The results of the study carried out by Leal *et al*.^17^ showed promising results regarding the biocontrol capacity of *Trichoderma atroviride* SC1 against infections of black‐foot pathogens during the propagation process of cv. Tempranillo. Other studies with *T. atroviride* SC1 have also shown a reduction in the incidence of several *Dactylonectria* and *Ilyonectria* species following *Trichoderma* treatment during the grapevine propagation process.^18^

One of the points of interest to consider when protecting vines with BCAs is the pH of the soil, which is one of the most influential factors in the communities of microorganisms that can be found in soils.^19^, ^20^ For this reason, the soil pH can become a limiting factor in effectiveness when applying *Trichoderma* as a BCA. In the study carried out by Carro‐Huerga *et al*.,^11^ a negative correlation was observed between the presence of *Trichoderma* and the soil pH. The lower the pH, the greater the presence of *Trichoderma* in the soils.

Another important factor for successful biocontrol is ensuring the survival of *Trichoderma* strains. The success of *Trichoderma* in the control of GTDs is highly dependent on environmental changes and soil properties,^21^ therefore it is of great interest to formulate these BCAs to extend their survival in the soil and to increase their efficacy as BCAs. Depending on the carrier materials used, microbial formulations can be prepared as granules, powders, or liquids.^22^ Bioformulations for bioinoculants are an important line of research in order to optimize application and efficacy under field conditions.^23^ Specifically, solid formulation has a great variety of compounds used as additives and carriers that can be implanted in a bioformulation.^24^ For example, Vermicompost‐based formulations have been shown to retain shelf life for up to 220 days at 30 °C.^25^ Other substances used as carriers provide sources of carbon and nitrogen (such as dextrose or glucose and peptone or tryptophan) nutrients necessary for *Trichoderma* growth.^26^ Wheat flour residues can serve as a carbon source for beneficial microorganisms. The advantage of including carbon sources like wheat flour residues in bioformulations is that they provide nourishment and support for the survival of microbes.^27^ In the case of algae, particularly blue‐green algae (cyanobacteria), they are used in *Trichoderma* bioformulates to enhance their efficacy and provide additional benefits for plant growth and soil health. These algae are an excellent source of nitrogen due to their high protein content (40–65%) and polysaccharides (5–10%).^28^, ^29^ Finally, hop residues have a nutritional composition that makes them a potential source of plant nutrients, with a carbon:nitrogen ratio of 13:1, which can serve as a source of carbon and nitrogen for *Trichoderma*.^30^ At present there are very few studies that make use of hop residues in bioformulates, although there are studies in which it is used in combination with *Trichoderma*.^31^, ^32^

This study aimed to select *Trichoderma* strains adapted to alkaline soil that also exhibit *in vitro* biocontrol potential against black‐foot disease pathogens and that can be adapted for their use in bioformulations. The specific objectives of this study were (i) to select and identify *Trichoderma* strains with the ability to grow in alkaline pH vineyard soils; (ii) to evaluate the ability of the best *Trichoderma* strains adapted to alkaline to control different pathogens causing black‐foot disease; and (iii) to analyze the viability of *Trichoderma* on vegetal residue‐based formulations.

## 2 MATERIALS AND METHODS

### 2.1 Fungal strains

The 47 *Trichoderma* isolates used in this study were selected from the collection of the Laboratorio de Diagnóstico de Plagas y Enfermedades Vegetales (Plant and Pest Diagnostic Laboratory) (LDPEV) (University of León, Spain). The isolates chosen were obtained from vine plots in different regions of Spain to have the maximum possible variability, as well as from different parts of the grapevine plant (Table 1).

**Table 1:** Information (code of *Trichoderma* given, origin of isolation, viticulture characteristics of the origin and PDO region isolated from) for the 47 *Trichoderma* isolates used in the alkaline pH growth assay

*Trichoderma* isolate code number	Isolated area	Characteristics	Region of origin
T065	Soil	Albarín white variety vineyard	PDO Leon
T066	Soil	Prieto Picudo variety vineyard	PDO Leon
T071	Soil	Tempranillo variety vineyard	PDO Leon
T081	Plant	Trunk of the Tinta de Toro variety	PDO Toro
T083	Plant	Vine shoot of the Tinta de Toro variety	PDO Toro
T087	Soil	Tinta de Toro variety vineyard	PDO Toro
T108	Plant	Dead grapevine, Prieto Picudo variety	PDO Leon
T109	Plant	Vine shoot of the variety Mencía	PDO Bierzo
T112	Plant	Vine bark of Tempranillo variety	PDO Toro
T117	Plant	Vine bark of Prieto Picudo variety	PDO Leon
T130	Plant	Vine bark of Mencía variety	PDO Bierzo
T131	Plant	Vine bark of Prieto Picudo variety	PDO Leon
T147	Plant	*Fagus silvatica*	Pirineos Mountains
T150	Plant	Trunk of the Tempranillo variety	Pago de Carraovejas Winery
T151	Plant	Trunk of the Cabernet Sauvignon variety	Pago de Carraovejas Winery
T154 (*Trichoderma carraovejensis*)	Plant	Trunk of the Tempranillo variety	Pago de Carraovejas Winery
T175	Soil	Prieto Picudo variety vineyard	PDO Leon
T177	Soil	Tinta de toro variety vineyard	PDO Toro
T179	Soil	Prieto Picudo variety vineyard	PDO Leon
T181	Soil	Tempranillo variety vineyard	PDO Leon
T187	Soil	Mencía variety vineyard	PDO Bierzo
T191	Soil	Prieto Picudo variety vineyard	PDO Leon
T193	Soil	Tempranillo variety vineyard	PDO Toro
T214	Plant	Bark of vine plant	Ossian Winery
T226	Plant	Tap root of vine plant	PDO Cangas del Narcea
T227	Soil	Vineyard	PDO Cangas del Narcea
T238	Soil	Vineyard	PDO Cangas del Narcea
T241	Plant	Neck of vine plant	PDO Cangas del Narcea
T254	Soil	Vineyard	Ossian Winery
T255	Soil	Vineyard	Ossian Winery
T263	Plant	Tap root of vine plant	PDO Cangas del Narcea
T265	Plant	Neck of vine plant	PDO Cangas del Narcea
T271	Plant	Neck of vine plant	Ossian Winery
T272	Plant	Trunk of vine plant	Ossian Winery
T275	Plant	Secondary root of vine plant	Ossian Winery
T280	Soil	Vineyard	Ossian Winery
T284	Plant	Neck of vine plant	Ossian Winery
T285	Plant	Tap root of vine plant	Ossian Winery
T292	Plant	Neck of vine plant	Ossian Winery
T294	Soil	Vineyard	Ossian Winery
T296	Soil	Vineyard	Ossian Winery
T297	Soil	Vineyard	PDO Cangas del Narcea
T300	Plant	Neck of vine plant	Ossian Winery
T302	Soil	Vineyard	Ossian Winery
T305	Plant	Inside the neck of the vine plant	Pago de Carraovejas Winery
T309	Soil	Vineyard	Ossian Winery
T310	Plant	Vine shoot of the variety Mencía	PDO Leon

Abbreviation: PDO, Protected Designation of Origin.

Five different strains of the fungi causing black‐foot disease of grapevine were used in the study. *D. novozelandica* (P150) and two strains of *D. torresensis* (P112 and P140) were isolated from young, symptomatic vine plants in the PDO Rueda region of Castilla y León, Spain. They were identified in the Laboratory of Instrumental Techniques of the University of León with multi‐locus sequence typing of four loci, internal transcribed spacer region of nuclear rDNA ITS1–5.8S–ITS2 (ITS), beta‐tubulin (TUB2), translation elongation factor 1‐alpha (TEF1), and histone 3 (HIS3). These isolates are part of the LDPEV collection.^33^ The other two species used, *D. alcacerensis* (BV‐1240) and *I. vivaria* (BV‐1924), were kindly provided by research group of Dr. David Gramaje from the Instituto de Ciencias de la Vid y del Vino, University of La Rioja, Spain.^14^

### 2.2 Analysis of the growth capacity at alkaline pH (8.5)

To analyze the growth capacity of the 47 *Trichoderma* isolates at pH of 8.5, an experiment was carried out under *in vitro* conditions. Fungal growth on *in vitro* adverse conditions was evaluated in 90‐mm Petri dishes with potato dextrose agar (PDA) medium (Sigma‐Aldrich Chemie GmbH, Steinheim, Germany). After autoclaving, NaOH was added until a pH of 8.5 was reached. Subsequently, mycelial discs of the 47 *Trichoderma* isolates obtained from 7‐day‐old cultures were plated into the Petri dishes with an alkaline PDA medium. Four replicates were performed for each *Trichoderma* isolate. Colony diameters were measured after 72 h.

### 2.3 Antagonism assays in dual cultures

The antagonistic capacity of the five *Trichoderma* isolates that showed the best growth at pH 8.5 (T071, T181, T255, T285, and T305) was evaluated *in vitro* by performing a dual culture assay against the pathogens causing black‐foot disease of grapevine. First, mycelial plugs (6 mm in diameter) of each strain were obtained from the edge of 7‐day‐old PDA culture plates grown at 25 °C. These mycelium plugs were placed at one end of a PDA (pH 8.5) Petri dish. At the same time, another 6‐mm diameter mycelial plug of *Trichoderma* was placed next to the pathogen at a distance of 55 mm. This was obtained from the edge of 7‐day‐old PDA culture grown at 25 °C. Four replicates were used for each *Trichoderma* strain, as well as a control in which only the pathogenic fungus was placed. The dual cultures were then incubated in the dark at 25 °C.

Pathogen growth radii were measured at 10 days after inoculation. The inhibition percentage caused by *Trichoderma* was calculated with the following equation^8^:

$$\text{growth inhibition}\;\left(\%\right)=\frac{\left(r1-r2\right)}{r1}\times 100$$

where *r*2 is the radius of the pathogen mycelium growth co‐inoculated with *Trichoderma* and *r*1 is the radius of the pathogen mycelium growth alone in the control plate.

### 2.4 Genome sequencing

The genome sequence of the T285 isolate was generated at Macrogen Inc. (Seoul, Korea; https://dna.macrogen.com) using an Illumina platform. Sequence assemblies were generated as previously described.^34^ The processed sequence reads were submitted to the Sequence Read Archive database at the National Center for Biotechnology Information (NCBI) under accession number SRR32453441 (Bioproject PRJNA1226781).

### 2.5 Phylogenetic analyses

Nucleotide sequences of 17 *Trichoderma* housekeeping (= HK) genes (Table S1) recovered from the genome of 26 *Trichoderma* species^34^, ^35^ were used to infer a *Trichoderma* species tree including strain T285.

The sequences of each gene of the 26 *Trichoderma* species used were individually aligned with the MUSCLE software implemented in MEGA X^36^ and then concatenated using the Sequence Matrix software.^37^ The resulting concatenated alignment was then subjected to maximum likelihood (ML) analysis as implemented in the program IQ‐TREE version 1.6.12.^38^ A second concatenated‐partitioned tree was constructed by selecting the best‐fit evolutionary nucleotide model for each gene deduced from the previous IQ‐TREE analysis. Finally, both concatenated (non‐partitioned and partitioned) alignments were subjected to ML analysis as implemented in IQTREE. Branch support was determined by bootstrap analysis using 1000 pseudoreplicates.^35^, ^39^ In addition, to assess the consistency of trees inferred from the 17 housekeeping genes, a gene concordance factor (gCF) analysis was performed as described by Minh *et al*.^40^ To support these studies, three other trees were inferred using partial amino acid sequences deduced from coding sequences of three housekeeping genes [*acl1* (ATP citrate lyase), *tef1* (translation elongation factor 1‐alpha), and *rpb2* (RNA polymerase 2nd largest subunit)], which were retrieved from different species of Jaklitsch *et al*.^41^ These sequences were aligned by MUSCLE software as implemented in MEGA X, and the trees were generated with the program IQTREE version 1.6.12. Branch support was assessed by a bootstrap analysis based on 1000 pseudoreplicates.

### 2.6 Growth rate trials and morphological characterization of T285

As described in Zanfaño *et al*.,^34^ the growth rate and optimum growth temperature of the strain T285 was determined on 90‐mm diameter Petri dishes using three different culture media: PDA, corn meal dextrose agar (CMD; 40 g of cornmeal, 20 g of glucose, 18 g of agar, 1 L of distilled water) and synthetic nutrient‐poor agar (SNA; 1 g of KH_2_PO_4_, 1 g of KNO_3_, 0.5 g of MgSO_4_, 0.5 g of KCl, 0.2 g of glucose, 0.2 g of sucrose, 18 g of agar, 1 L of distilled water) at 20, 25, 30 and 35 °C. Plugs of 6‐mm diameter were taken from the edge of 7‐day‐old PDA plates and placed approximately 1 cm from the edge of the Petri dishes. Four replicates were made for each temperature. The diameters of the colonies were measured after 24, 48, 72, 96 h and 7 days. The time when the mycelium completely covered the surface of the plate was also recorded in this assay. The morphological characteristics of the colonies, such as appearance, color, and spore production, were recorded at the same time.

To evaluate its microscopic morphology, the isolate T285 was cultured on PDA and SNA for 72–96 h at 25 °C. A total of 50 conidia and phialides were measured to obtain a representative sample. The following characters were measured: conidia length and width, length of phialides, and width of phialides at the widest point. Images were taken using a Nikon Eclipse E600 microscope connected to a Nikon DS‐Fi3 digital camera.

Spore production was quantified using four biological replicates of *Trichoderma* cultures grown on PDA medium. A mycelial plug (5 mm diameter) was taken from the edge of a 7‐day‐old culture and used for point inoculation on the center of a 90‐mm Petri dish for each repetition. The inoculated plates were then incubated in the dark at 25 °C for 7 days to allow for optimal sporulation. After the incubation period, spores were harvested by adding 10 mL of sterile distilled water to each dish and gently scraping the surface of the colony with a sterile glass rod to detach the conidia. The resulting suspension was collected and passed through two layers of sterile muslin filter cloth to remove hyphal fragments and agar debris. The filtered spore suspension was thoroughly mixed, and an aliquot was transferred to a Neubauer improved hemocytometer. Spore concentration was determined by direct microscopic counting under a light microscope at 400× magnification. Final spore density was expressed as the mean number of conidia per milliliter, calculated from at least five independent fields of view per replicate.

### 2.7 Vegetal residue‐based formulation

For this experiment, *Trichoderma* strain T285 was selected from the previous dual culture assay due to its highest capacity for growth and to control a black‐foot disease fungus. This experiment consists of evaluating the durability of three formulations made with T285. To obtain a balanced formulation, it is necessary to include different concentrations from different compounds that are related as follows. At least one source of carbon and a source of nitrogen for stimulating a faster growth of *Trichoderma*.^26^ Also, an elicitor compound is important to add to the formulation with the aim of stimulating hydrolytic production.^42^ Moreover, excipients are needed included to enhance good performance of a proper formulation.^22^ Finally, according to the bibliography, the concentration of *Trichoderma* spores in most products applied during the last two decades has ranged from 1 × 10^5^ and 1 × 10^10^ CFUs/mL.^1^, ^22^ All these steps lead to the proper formulation of a *Trichoderma*‐based product and we hypothesize better performance under field conditions in comparison to direct application from water‐based spores. Three different formulations were carried out, each prepared with a different residue as carbon and nitrogen sources: one based on wheat flour residues (T1), another one composed of hop residues (T2), and the last one made of algae residues (T3). Wheat flour residues are by‐products obtained from the milling of wheat grains that do not meet the quality standards required by the flour industry. Hop residues originate from the harvest remains of the plant. In the case of algal residues, they derive from the species *Sargassum muticum* and are obtained after processing in the cosmetic and pharmaceutical industries. The base products were mixed with kaolin, peptone, and chitosan. Isolate T285 was incorporated into the formulations at a concentration of 2 × 10^7^ CFU/mL.

**Figure 1:**
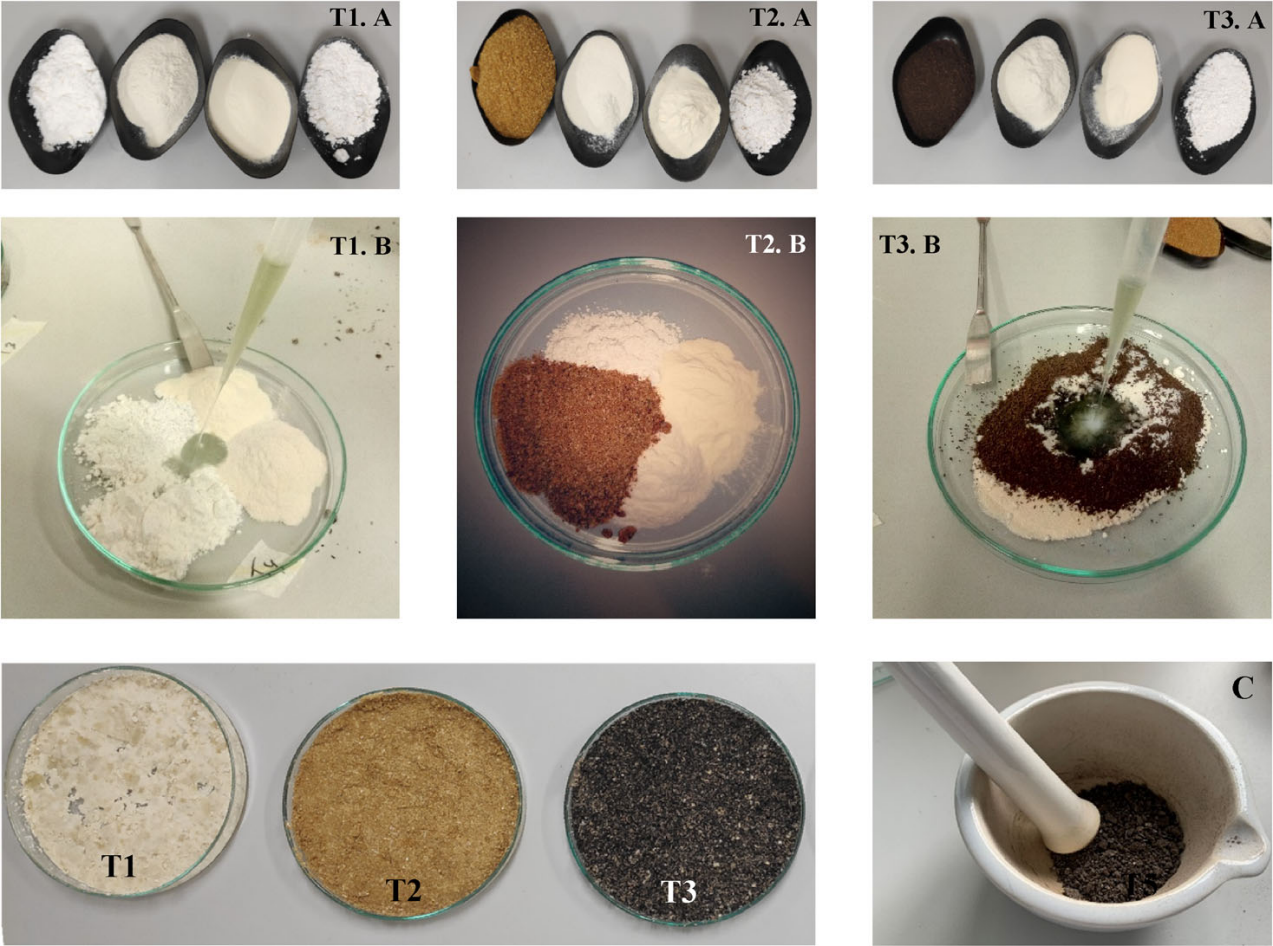
Formulation development process. T1, T2, and T3 correspond to the wheat flour, hops, and algae residue‐based formulations, respectively. The images labeled A show the different compounds that make up each formulation. The images labeled B show the mixing process of all the compounds of the different formulations. The lower T1, T2, and T3 images show the final paste generated from each treatment before drying. Image C shows the process of breaking and crushing the formulated paste.

The preparation of these formulations was carried as follows: first, hop and algae residues were ground in a grinder. Then, these two products together with the wheat flour residues were sieved with a 150‐μm sieve. Subsequently, all products used in the formulations, except chitosan and peptone, were autoclaved at 121 °C for 15 min. Afterward, mixtures of each treatment were prepared in glass Petri dishes. The concentration of wheat flour, hops, and algae residues was 29% (w/w). For kaolin, peptone, and chitosan, a concentration of 14% (w/w) each was used. The spore solution of T285 was the last product added to the mixture, reaching a final concentration of 29% (w/w), forming a paste that was left to dry for 24 h in a flow hood. Finally, once the mixture was dry, it was broken up using a spatula and a mortar until a very fine powder was obtained (Fig. 1).

To evaluate the durability of formulations, the presence of *Trichoderma* was analyzed on the day of formulation (D0), 30 days after formulation (D30), and 90 days after formulation (D90). During this time, the formulations were kept at 4 °C. Refrigeration at 4 °C is commonly used to evaluate the shelf‐life of microbial formulations because this temperature markedly slows metabolic activity and physicochemical degradation processes, thereby helping to preserve spore viability over extended storage periods. Several studies assessing *Trichoderma* and other biocontrol formulations have used refrigerated storage (4 °C) and periodic sampling at 30, 60, and 90 days to monitor viability and antagonistic activity, making storage at 4 °C an established method for durability testing of such formulations.^43^, ^44^ In the laboratory, a sample of each formulation was inoculated per triplicate in Petri dishes with Rose Bengal‐chloramphenicol agar (Conda Laboratory, Madrid, Spain) medium to count colony‐forming units (CFU) per gram of formulated product.

### 2.8 Formulations under field conditions

Vegetal residue‐based formulations were evaluated under field conditions. Two experiments were carried out. The first took place in May 2024, while the second took place in April 2025. Both experiments were conducted in the same vineyard but in different areas. The experiments consisted of a randomized split‐plot design of three replicates × five treatments in 2024 and 10 replicates × five treatments in 2025. The five treatments consisted of the three formulations of T285 based on wheat flour residues (T1), hop residues (T2), and algae residues (T3), a positive control with T285 (T4), and a negative control (T5) in which *Trichoderma* was not inoculated. For the controls (T4 and T5), the same formulations were prepared as for the residues (T1, T2, and T3) but the residues were replaced by talc (inert material) and in the case of the positive control, T285 was added at a concentration of 2 × 10^7^ spores/mL, and for the negative control, autoclaved distilled water was added.

**Figure 2:**
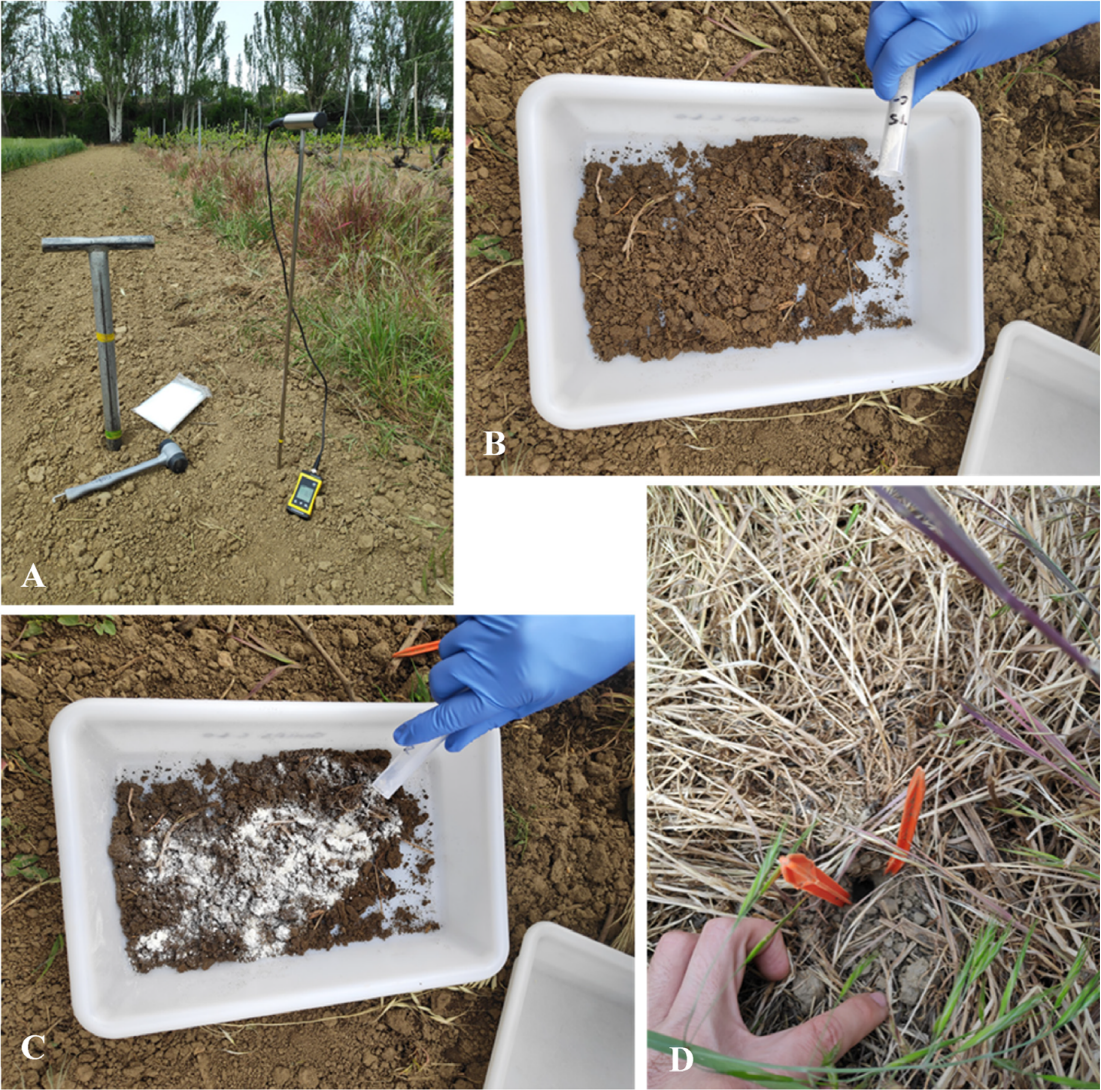
Soil incorporation of formulations. (A) extraction of soil volume corresponding to 30 cm depth. (B) Soil sample taken from the plot. (C) Mixing of the soil sample with the corresponding formulation. (D) Deposition zone of the soil‐formulated mixture and identification of the zone for subsequent sampling.

The study was carried out in the experimental vineyard of the Escuela de Ingeniería Agraria y Forestal of the University of León. Using a soil sampler probe, the volume of soil corresponding to a depth of approximately 30 cm was extracted and mixed with 14 g of each of the treatments. The soil‐formulated mixture was poured back into the hole made in the vineyard soil and left for 7 days (Fig. 2). Soil moisture and temperature measurements were also taken at various points when the treatments were applied. After this time, the presence of *Trichoderma* in the soil to which the formulation had been added was analyzed. A sample was taken from the soil where the different formulations had been applied. In the laboratory, the sample was dried out at room temperature until it was totally dry. After that, 1 g of soil was diluted in 10 mL of autoclaved distilled water in a sterile flask and it was placed in an orbital shaker for 1 h. Then, serial dilutions were performed and 100 μL was poured into Petri dishes with Rose Bengal‐chloramphenicol agar medium. Two replicates of each dilution were performed. Colony counts were converted into CFUs per gram of dry soil. Meteorological data were also obtained for the area during the test periods. For this, the Mansilla Mayor meteorological station from the INFORIEGO weather station network (Junta de Castilla y León, Government of Spain) was used. To identify *Trichoderma* spp. under field conditions, cultural characteristics were first assessed, followed by microscopic examination according to Harman and Kubicek.^45^ Because morphological traits alone are insufficient to reliably discriminate species within the genus, isolates were reported collectively as ‘*Trichoderma* spp. isolation’.

### 2.9 Statistical analysis

All the tests carried out were analyzed using IBM SPSS® statics 21 (IBM Corp. Armonk, NY, USA). This software was used for the statistical analyses as follows. First, it was checked if they had a normal distribution using Shapiro–Wilk and Kolmogorov–Smirnov tests, depending on *n* values <50 or *n* values >50, respectively. Then the homogeneity of variances was evaluated using Levene's and Kruskal–Wallis test and one‐way analysis of variance (ANOVA). In the case of ANOVA, a *post hoc* test (Tukey, *P* ≤ 0.05) was performed to establish differences between groups.

## 3 RESULTS

### 3.1 Analysis of the growth capacity at alkaline pH (8.5)

The growth performance of the 47 *Trichoderma* isolates assessed 72 h after inoculation showed considerable variation in colony diameter, ranging from 14.00 ± 1 mm (T238) to 72.88 ± 1 mm (T285), corresponding to more than a fivefold difference between the smallest and largest colonies. Isolate T285 exhibited the greatest growth under alkaline pH conditions, closely followed by isolate T305 (72.00 ± 1 mm), with no significant differences observed between them. Isolates with colony diameters greater than 65 mm included T071 (71.00 ± 1 mm), T181 (68.25 ± 1 mm), T255 (67.13 ± 1 mm), and T302 (65.13 ± 1 mm), all of which showed significantly higher growth compared with the remaining *Trichoderma* isolates. At the opposite extreme, isolate T238 exhibited the lowest growth, with an average colony diameter of 14.00 ± 1 mm. Three other isolates also displayed poor growth, not exceeding 20 mm in diameter: T310 (17.25 ± 1 mm), T109 (17.38 ± 1 mm), and T112 (18.00 ± 1 mm) (Table 2).

**Table 2:** Mean growth diameter of 47 *Trichoderma* isolates in potato dextrose agar (PDA) plates of alkaline medium (pH = 8.5) 72 h after inoculation (four replicates).

Isolated	Growth diameter (mm)	Significance
T285	72.88	*a*
T305	72.00	*a*
T071	71.00	*ab*
T181	68.25	*abc*
T255	67.13	*abcd*
T302	65.13	*bcde*
T175	64.88	*bcde*
T191	64.13	*cde*
T177	64.00	*cde*
T296	62.13	*cdef*
T083	61.38	*defg*
T254	60.13	*efgh*
T130	60.13	*efgh*
T081	60.00	*efgh*
T066	59.50	*efghi*
T131	59.00	*efghij*
T154	57.13	*fghijk*
T263	56.13	*fghijk*
T226	55.50	*fghijkl*
T065	55.25	*ghijkl*
T280	54.00	*hijklm*
T297	53.63	*hijklmn*
T179	53.25	*ijklmn*
T272	52.50	*jklmno*
T292	52.00	*klmno*
T265	51.63	*klmno*
T271	50.88	*klmno*
T150	50.63	*klmnop*
T147	48.88	*lmnopq*
T214	48.50	*mnopqr*
T294	47.13	*nopqr*
T300	46.00	*opqrs*
T108	44.13	*pqrst*
T087	43.00	*qrst*
T193	42.13	*rst*
T309	39.63	*stu*
T151	39.25	*tu*
T187	34.13	*uv*
T275	32.13	*v*
T284	31.38	*v*
T241	29.00	*vw*
T227	27.50	*vw*
T117	22.50	*wx*
T112	18.00	*xy*
T109	17.38	*xy*
T310	17.25	*xy*
T238	14.00	*y*

Different letters indicate significant differences between *Trichoderma* isolates. Tukey test (*P* ≤ 0.05).

### 3.2 Antagonism assay in dual cultures

**Figure 3:**
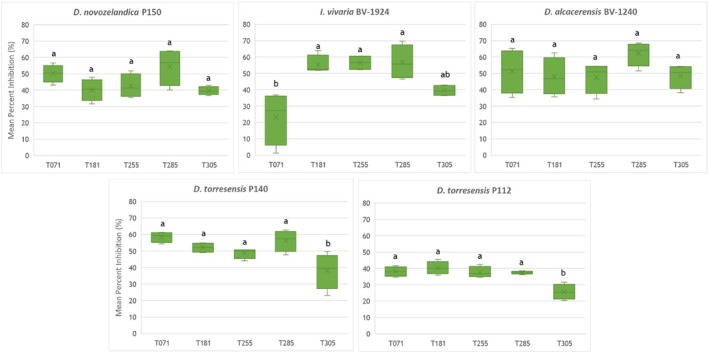
Mean percentage inhibition of radial growth of *Trichoderma* isolates against pathogens P150, BV‐1924, BV‐1240, P140, and P112 measured after 10 days. The data were calculated from four replicates for each pathogenic strain. Bars represent standard errors of the mean. Different letters indicate significant differences between *Trichoderma* isolates. Tukey test (*P* ≤ 0.05).

The percentages of inhibition of the *Trichoderma* isolates from this study against the pathogens that cause black‐foot disease are shown in Fig. 3. In the case of the pathogen P150, the average inhibition values ranged from 39.62% for strain T305 to 54.43% for T285, with no significant differences observed among the different *Trichoderma* isolates. BV‐1924 growth was inhibited at the highest level by T285 (56.84%), with no significant differences compared to T181, T255, and T305. The lowest inhibition value for this pathogen was recorded by T071 (23.18%), showing significant differences compared *versus* T181, T255, and T285. Not one of the *Trichoderma* isolates used showed significant differences in their inhibition against the pathogen BV‐1240, with values ranging from 47.60% to 62.18%. For P140, significant differences were observed only between T305 (37.96%) and the rest of the *Trichoderma* isolates, which exhibited inhibition percentages ranging from 48.71% to 58.54%. Finally, P112 presented the lowest inhibition values caused by the *Trichoderma* isolates, with significant differences observed only between T305 (25.72%) and the other isolates, whose inhibition values ranged from 37.40% to 40.45%. In Fig. S1, you can see how the different pathogens are confronted with T285.

### 3.3 Phylogenetic analyses

**Figure 4:**
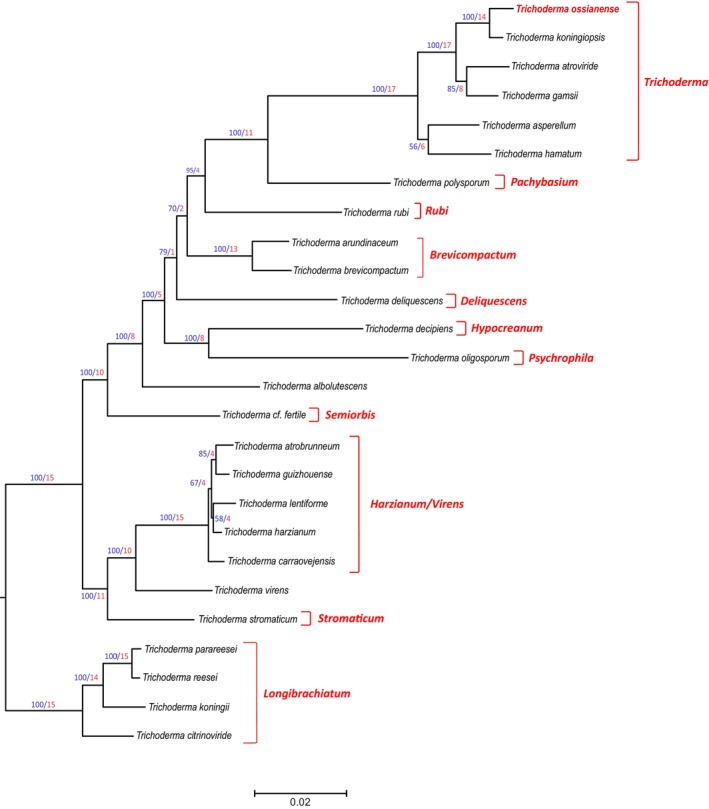
Phylogenic tree generated by the maximum likelihood analysis using concatenated sequences of 17 housekeeping genes of the genus *Trichoderma*. Sequences of housekeeping genes from *Trichoderma ossianense* T285 used for this study were deposited at the DDBJ/ENA/GenBank database under accession numbers indicated in Table S1. Numbers on branches are bootstrap values in percentage (blue type) based on 1000 pseudoreplicates and gene concordance factors (gCF, red type). gCF values indicate the number of each of the 17 independent trees that show the same branch, e.g. a value of 15 in a branch means that 15 out of the 17 individual trees show the same branch illustrated in the tree included in this figure. Lineage names, as previously described,^3^, ^38^ are indicated in red type at the right of the tree.

The 26 species of *Trichoderma* included in the phylogenetic analysis fall into 11 previously described lineages.^3^, ^35^ To determine the position of isolate T285, a species tree was inferred based on a maximum likelihood analysis of concatenated alignments of 17 housekeeping genes that were retrieved in previous work^34^, ^35^ as well as from the genome sequence of strain T285. In the resulting phylogenetic tree (Fig. 4), isolate T285 mapped on an individual branch in the *Trichoderma* clade, together with the species *T. koningiopsis*, *T. atroviride*, *T. gamsii*, *T. asperellum*, and *T. hamatum*. This branch yields a bootstrap value of 100 and a gCF of 14. These data support the assignment of isolate T285 to a new species, which has been named *Trichoderma ossianense* in reference to the name of the winery where it was isolated, Ossian. In addition, the structure of the phylogenetic tree obtained was consistent with the multispecies phylogenies previously reported for other *Trichoderma* species combinations.^3^, ^35^, ^41^

To support the previous analysis, three genetic markers (*acl1*, *tef1*, and *rpb2*) were used to construct three secondary trees (Figs [Link], [Link]). Phylogenetic analysis was performed using fragments of the *acl1*, *tef1*, and *rpb2* genes from a wide variety of isolates, resulting in trees consistent with the main tree of 17 housekeeping genes (Fig. 4). These three secondary trees yielded bootstrap values on the *T. ossianense* branch higher than 94%.

### 3.4 Growth rate trials

**Figure 5:**
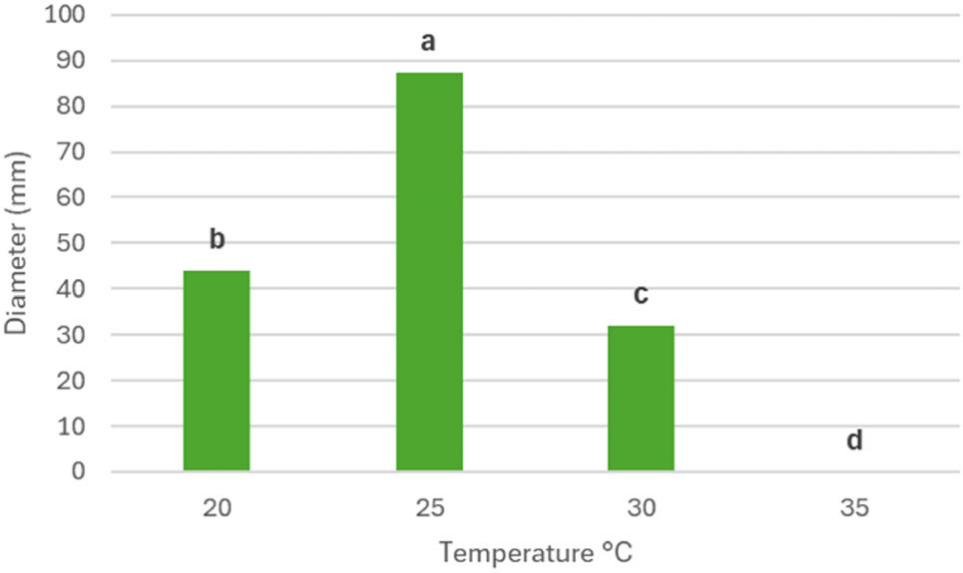
Growth rates of *Trichoderma ossianense* at 20, 25, 30 and 35 °C in potato dextrose agar after 96 h of incubation; Tukey test (*P* ≤ 0.05).

To determine the optimal growth temperature of *T. ossianense*, the results obtained from the growth rate assay on PDA medium after 96 h of incubation were analyzed. The results are shown in Fig. 5. The optimal growth temperature for *T. ossianense* was 25 °C, with a mean colony diameter of 87.25 ± 1 mm, which is significantly higher than the diameters reached at 20 (44 ± 1 mm), 30 (32 ± 1 mm), and 35 °C (0 ± 1 mm).

### 3.5 Taxonomy

**Figure 6:**
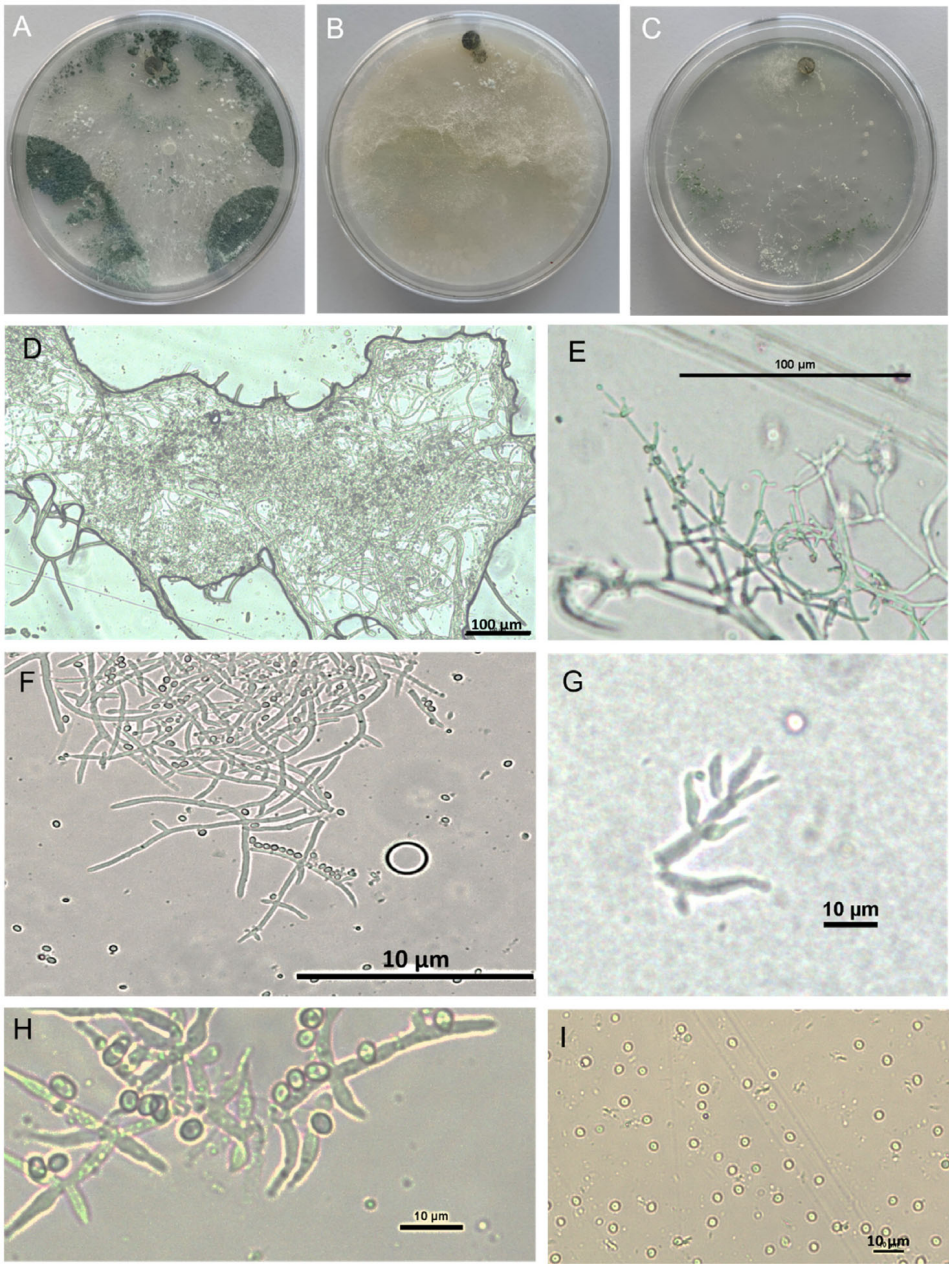
Observation of *Trichoderma ossianense*. (A) Cultures after 7 days at 25 °C on potato dextrose agar (PDA) medium. (B) Cultures after 7 days at 25 °C on corn meal dextrose agar (CMD) medium. (C) Cultures after 7 days at 25 °C on synthetic nutrient‐poor agar (SNA) medium. (D) Pustules formed after 7 days on PDA. (E) Mycelium with conidiophores and phialides on PDA. (F) Visualization of eidamia‐like morphology of conidiophores on PDA. (G) Detail of conidiophores lageniform, slightly swollen in the middle when more crowded, straight, on SNA medium. (H) Phialides and conidia on PDA medium. (I) Detail of conidia on PDA.

*T. ossianense*: L. Zanfaño, G. Carro‐Huerga, S. Gutiérrez. P.A. Casquero Luelmo. sp. nov. (Fig. 6).

*Etymology*: Referring to the winery where the isolate was obtained: ‘Ossian vides y vinos S.L.U.’. MycoBank registration number MB860609.

*Typus*: Spain, Castilla y León, Segovia, Nieva, tap root of vine plant (*Vitis vinifera*), L. Zanfaño (NCBI accession number SRR32453441) (Bioproject PRJNA1226781).

*Notes*: This study isolated *T. ossianense* from the tap root of a vine plant. It was differentiated from other species through a phylogenetic analysis of 17 housekeeping gene sequences from 26 different *Trichoderma* species, and its morphology has been described.

Moderate growth of colonies. Initially, a light mycelium is formed, which sporulates randomly on the plate without completely covering it with spores after 7 days of incubation. No distinctive odour. Conidia form compact, confluent pustules at the colony's margins, with little aerial mycelium present. The mycelium consists of branched, septate, and hyaline hyphae. Conidiophores are paired with single lentiform phialides or in groups of two or three (9.9−)10.8–11.3(−12.9) × (2.1−)2.3–2.8(−3.4) μm, length/width ratio. The conidia belong to the blastoconidia group and have a slightly oval, globose shape with smooth margins, (3.8−)4.1–4.4(−4.7) × (2.7‐)3.0–3.4(−3.8) μm, length/width ratio.

*Culture characteristics*: Optimum growth temperature is 25 °C. On PDA medium, the 90 mm diameter plate is covered after 5 days. On CMD it reaches a radius of 42 ± 1 mm after 7 days and on SNA it covers the plate after 7 days. At 20 °C, the fungus covers the PDA plate in 6 days, on CMD it reaches a radius of 36 ± 1 mm after 7 days, and on SNA it reaches 60 ± 1 mm after 7 days. At 30 °C, it can only develop on PDA and reaches a radius of 53 ± 1 mm after 7 days. At 35 °C the fungus is unable to grow on any of the used media. After 7 days of incubation in PDA at 25 °C, several colonies of deep‐green spores form alongside areas of light, whitish mycelium. In CMD, no spores form; only dense, yellowish‐tinged mycelium is visible. In SNA, a very weak mycelium forms with some sparse deep‐green spores. The production of spores in PDA at 7 days was 1.59 × 10^8^ spores/mL.

### 3.6 Vegetal residue‐based formulation

**Figure 7:**
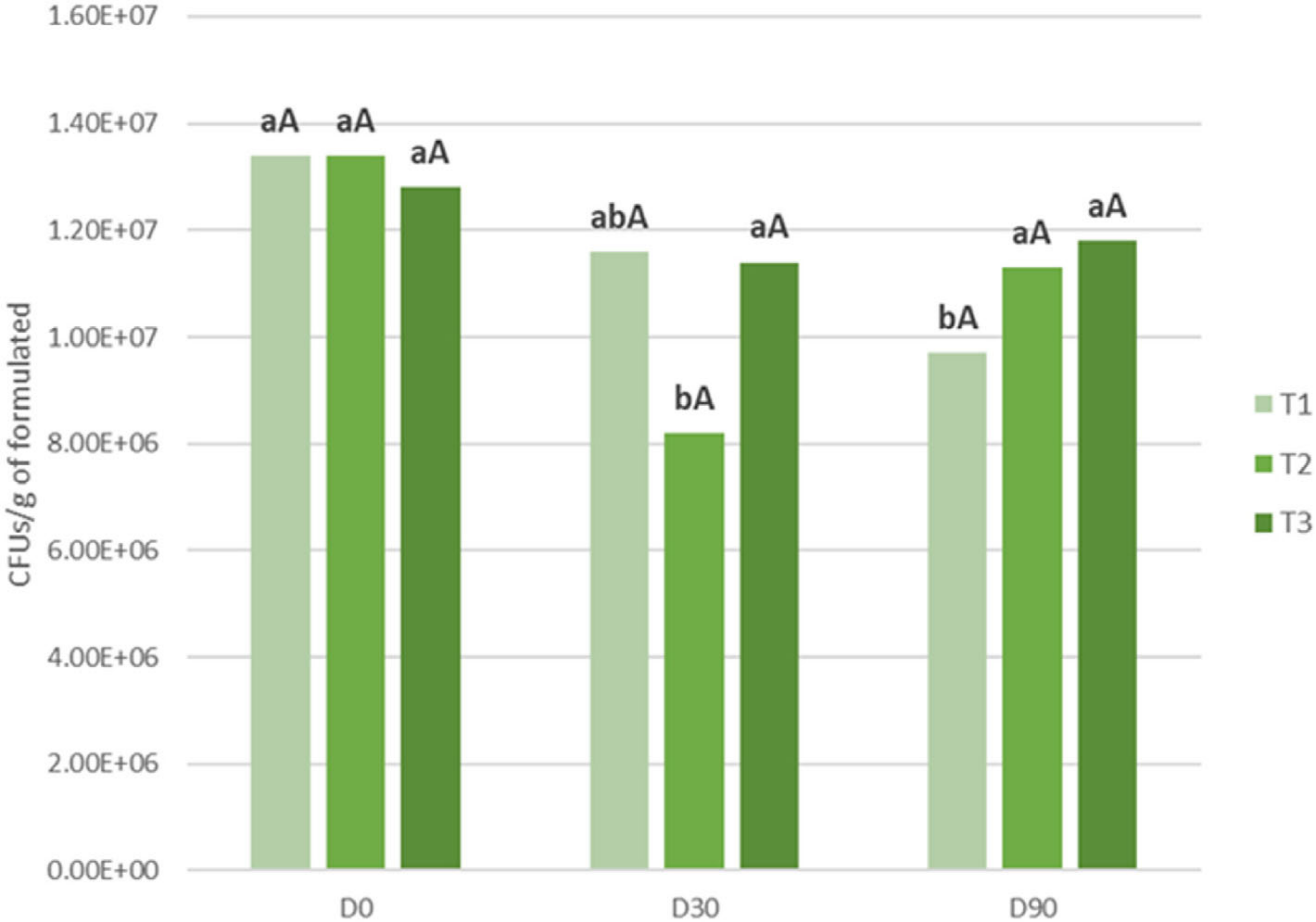
Concentration of T285 (CFUs/g) in different formulations at three different times: D0 (after formulation), D30 (after 30 days at 4 °C) and D90 (after 90 days at 4 °C). T1, formulation based on wheat flour residues; T2, formulation based on hop residues; T3, formulation based on algae residues. Different lower case letters indicate significant differences between days for the same treatment and different capital letters indicate significant differences between treatments on the same day. Tukey test (*P* ≤ 0.05).

Among the strains previously evaluated, T285 exhibited the most favorable results, demonstrating both robust growth under alkaline pH conditions and effective suppression of the pathogens associated with grapevine black‐foot disease. The results show a significant decrease in spore concentration for T1 between D0 (1.34 × 10^7^ CFU/g) and D90 (9.17 × 10^6^ CFU/g). T2 shows a significant decrease between D0 (1.34 × 10^7^ CFU/g) and D30 (8.2 × 10^6^ CFU/g), with the number of CFU increasing again at D90 (1.13 × 10^7^ CFU/g) also significantly different from D30. At T3, no significant differences were observed between D0 (1.33 × 10^7^ CFU/g), D30 (1.14 × 10^7^ CFU/g) and D90 (1.18 × 10^7^ CFU/g), with the spore concentration remained stable during this period. No significant differences were observed between treatments over the same period (Fig. 7).

### 3.7 Formulations under field conditions

**Figure 8:**
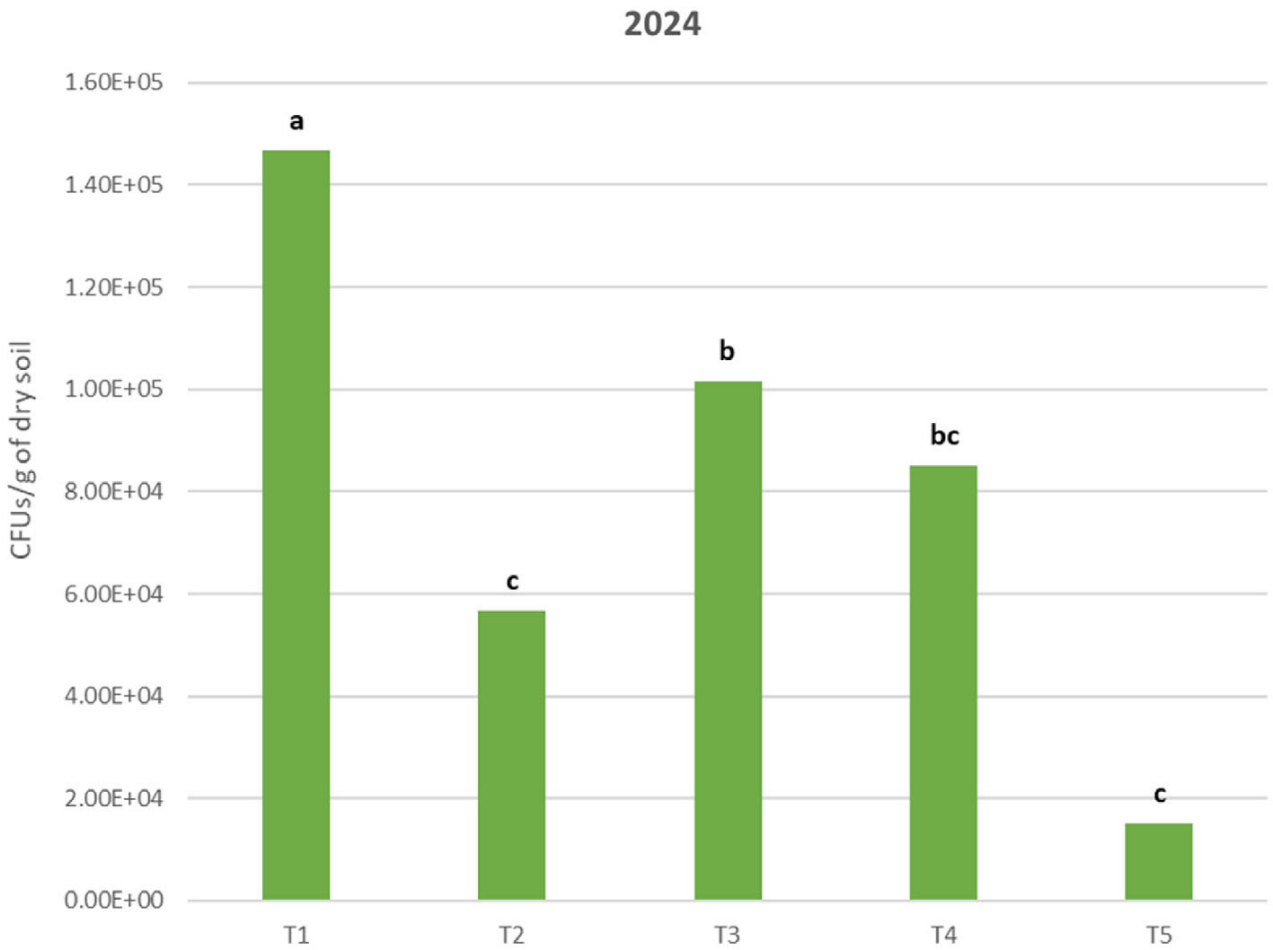
Identification of *Trichoderma* spp. isolation from different treatments in vineyard soil 7 days after inoculation during the 2024 trial. T1, formulation based on wheat flour residues; T2, formulation based on hop residues; T3, formulation based on algae residues; T4, positive control; T5, negative control. Different lower case letters indicate significant differences between treatments. Tukey test (*P* ≤ 0.05).

**Figure 9:**
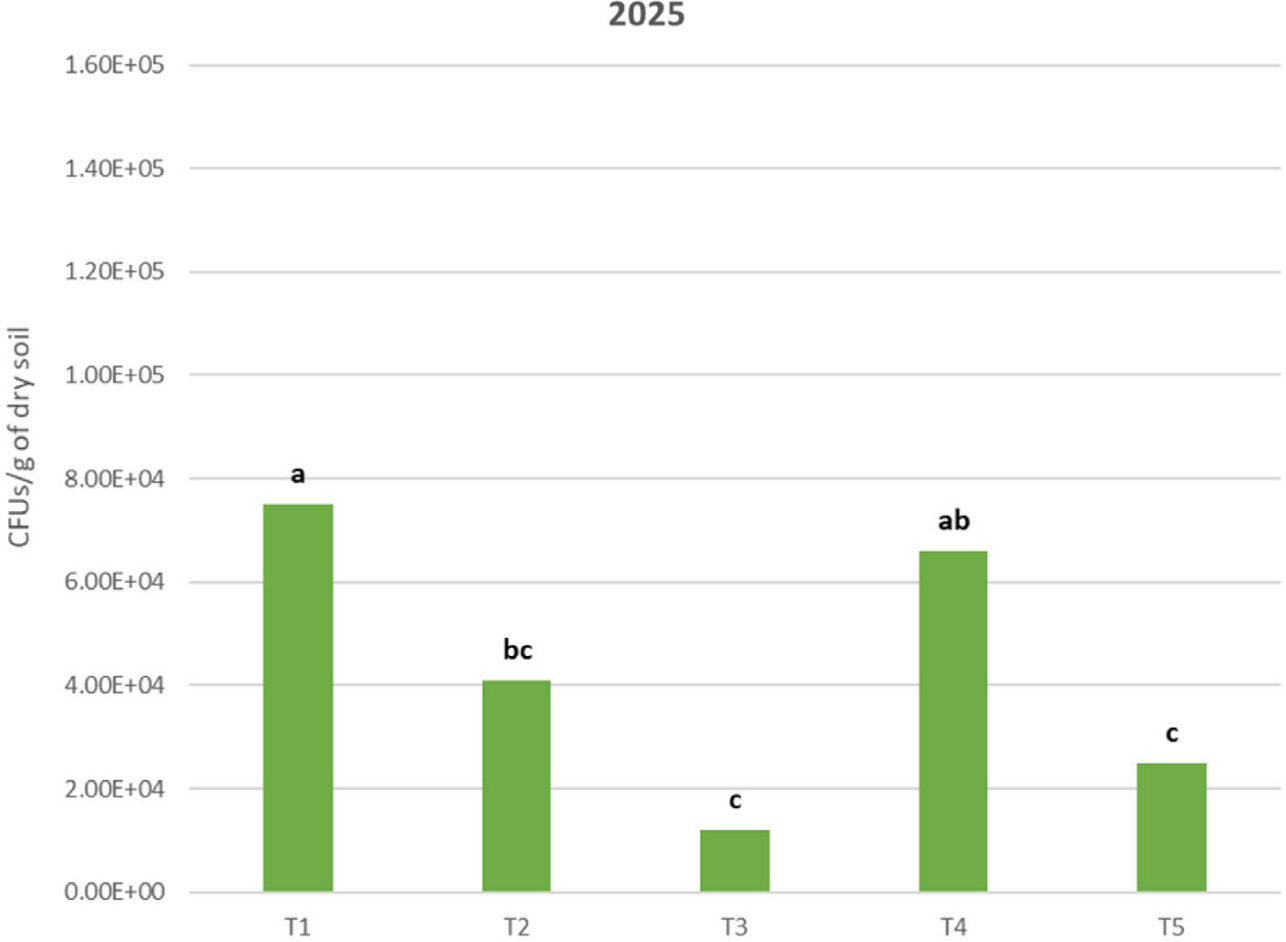
Identification of *Trichoderma* spp. isolation from different in vineyard soil 7 days after inoculation during the 2025 trial. T1, formulation based on wheat flour residues; T2, formulation based on hop residues; T3, formulation based on algae residues; T4, positive control; T5, negative control. Different lower case letters indicate significant differences between treatments. Tukey test (*P* ≤ 0.05).

Re‐isolation analysis of *Trichoderma* spp. from soil showed the following results. In 2024, a concentration of 1.47 × 10^5^ CFU/g in treatment T1 exhibited a significantly higher spore concentration than the other treatments. This was followed by T3, which reached a concentration of 1.02 × 10^5^ CFU/g. T3 differed significantly from T2 and T5 but did not differ from T4 (8.50 × 10^4^ CFU/g). T2 exhibited a concentration of 5.67 × 10^4^ CFU/g, which is significantly different from T1, T3, and T5. Finally, the negative control, T5, obtained a concentration of 1.50 × 10^4^ CFU/g, which was significantly lower than the other treatments (Fig. 8). In the 2025 trial, treatment T1, with a concentration of 7.50 × 10^4^ CFU/g, showed a significantly higher spore concentration than treatments T2, T3, and T5. This was followed by T4, which had a concentration of 6.60 × 10^4^ CFU/g, with no significant difference compared to T1 and T2, which had a concentration of 4.10 × 10^4^ CFU/g. T5 (2.50 × 10^4^ CFU/g) and T3 (1.20 × 10^4^ CFU/g) had the lowest re‐isolation values, exhibiting significant differences compared to T1 and T4 but not to T2. The detection of *Trichoderma* in T5 (negative control) is likely possible due to the presence of native *Trichoderma* in the soil where the test was carried out (Fig. 9). At the time of establishing the trial, the average soil moisture and temperature were 18.8% and 12.5 °C, respectively, in 2024, and 15.54% and 13.3 °C, respectively, in 2025. During the trial, precipitation was recorded at 3.04 mm in 2024 and 27.92 mm in 2025. The average ambient temperature was recorded at 13.9 °C in 2024 and 9.5 °C in 2025. The two trials were analyzed independently due to the variability in soil and climatic conditions, which influence the evolution of the formulations in the soil. All data were expressed as *Trichoderma* spp. isolation.

## 4 DISCUSSION

The aim of this work was to search for a *Trichoderma* strain adapted to alkaline soils such as those of some wine‐growing regions of Spain. It was also intended that the strain chosen would have the ability to control *in vitro* the pathogens causing black‐foot disease in vineyards. The development of many *Trichoderma* strains belonging to the LDPEV collection (University of León), all of them isolated from different parts of vineyards all over Spain, was studied. Following this study, five strains were selected, T071, T181, T255, T285, and T305, which showed the greatest ability to grow in alkaline soils. Among these strains, T285 and T305 were the best adapted to alkaline soils, with T285 exhibiting the strongest inhibitory capacity against the pathogens responsible for grapevine black‐foot disease. Three different formulations were developed with strain T285 and their durability was studied after different storage periods, as well as their persistence under field conditions. Finally, phylogenetic studies showed that isolate T285 was a new species of the genus *Trichoderma*.

This research work has also led to the description of a new indigenous *Trichoderma* species, isolate T285, now called *T. ossianense*. This *Trichoderma* strain was isolated from the root of *Vitis vinifera cv*. Verdejo at Winery Ossian in Nieva (Segovia, Spain) (Tables 1 and S1).

Several new *Trichoderma* species have been discovered recently.^46^ However, the number of new *Trichoderma* species described is still increasing, most probably because fungal soil communities have not been widely investigated yet. The phylogenetic position of these novel species was determined by analyzing only the concatenated sequences of the second largest nuclear RNA polymerase subunit encoding gene (*rpb2*) and the translation elongation factor 1‐alpha encoding gene (*tef1*). In the current study, we also used a large number of housekeeping genes (17) to describe a new species.^34^ Thus, the identification of new species is highly accurate. This newly identified *Trichoderma* species is closely related to *T. koningiopsis*, which may explain certain similarities in their morphology and development. Thanks to the detailed descriptions provided by Samuels *et al*.^47^ it has been possible to compare these two species. For instance, the optimal growth temperature of *T. koningiopsis* is 30 °C, whereas that of *T. ossianense* is slightly lower, at 25 °C. Regarding morphology, the phialides and conidia of *T. ossianense* are somewhat larger in size. Altogether, these observations confirm that although *T. ossianense* is phylogenetically very close to *T. koningiopsis*, they can be considered distinct species.

Soil pH is known to act as a predictor of the bacterial and fungal composition of the soil, strongly influencing these populations.^20^, ^48^ pH is one of the main factors affecting both the activity of *Trichoderma* and the different pathogenicity factors of some microorganisms, therefore it is very important to look for *Trichoderma* strains adapted to their optimum pH to ensure their good performance as a BCA.^49^ Some studies have shown that many *Trichoderma* isolates have their mycelial growth optimum at pH 4.0 but can vary over a pH range of 2.0–6.0. It has also been shown that the activity of some *Trichoderma* extracellular enzymes occurs over a wider range of pH values than those that allow mycelial growth.^50^ Analyses of the influence of physical and chemical soil parameters on fungal populations have shown a negative correlation between pH and fungal diversity.^51^ However, some species, such as *Ilyonectria liriodendra*, grow well at a pH of between 5 and 11, inhibiting their development at pH 12 and below 5.^52^

Black‐foot disease is one of the major problems in vineyards. The disease has been associated mainly with *Ilyonectria* and *Dactylonectria* species. Recent studies have shown an increased diversity of *Ilyonectria*‐like fungi in young vineyards.^53^ Currently, *Trichoderma* has been shown to exhibit variable efficacy against various pathogens causing grapevine black‐foot disease. The study by Van Jaarsveld *et al*.^54^ revealed that inhibition values can vary significantly depending on the *Trichoderma* strain used, with some cases reaching 100% inhibition. Similar to this study, macroscopic interactions were also observed between *Trichoderma* and the black‐foot disease pathogens, with total or partial overgrowth (Fig. S1). In some cases, this effect was associated with sporulation of *Trichoderma*. This highlights the importance of identifying indigenous *Trichoderma* strains that can control the pathogens responsible for grapevine black‐foot disease. In this study, all *Trichoderma* strains exhibited high levels of pathogen inhibition after 10 days of incubation at pH 8,5. In all cases, the fungi colonized the entire Petri dish and grew on top of the pathogen, thus preventing its development. T285 exhibited the strongest inhibitory activity against BV‐1924 and BV‐1240, one of the most virulent strains studied.^14^ All these values are very encouraging, and in the future, different *Trichoderma* strains could be used in combination to combat the different pathogens that cause black‐foot disease of grapevine.^17^

*Trichoderma* formulations are of great importance as they can delay the decline of the fungal population by protecting the conidia and providing nutrients to stimulate their growth. To ensure their efficacy after application in the field, products formulated with microorganisms must be durable and stable. For this reason, the products that constitute this formulation must be carefully selected, and their storage stability must be studied beforehand. From the perspective of circular economy and bioeconomy, the reuse of agrifood residues can help solve serious environmental problems such as soil contamination, degradation, erosion, and climate change.^55^ In our study, the stability at the level of *Trichoderma* concentration remained constant for 90 days in the T3 formulation, made with algae residues. However, formulations T1 and T2, formulated with wheat flour residues and hops residues, respectively, showed lower stability due to the fluctuation in the concentration of *Trichoderma* during the study period. One of the factors influencing the stability of these products is the storage temperature. Thus, lower storage temperatures generally extend shelf life, for example, some products can last 2 years at 4 °C but only 6–8 months at 15–25 °C.^22^ Another factor is the type of formulation. Generally, solid formulations tend to be more stable than liquid ones.^56^ The residues used (wheat flour, hops,^31^ and algae^57^) provide essential nutrients to *Trichoderma*. In addition, compounds such as chitosan, a polysaccharide capable of triggering host defense mechanisms against diseases like *Diplodia seriata* and *Phaeomoniella chlamydospore*,^58^ can enhance the biocontrol potential of *Trichoderma* against certain GTDs. Even some inert materials, such as kaolin, which provide structural support and protect the formulation from environmental stress,^59^, ^60^ contribute to its stability. Collectively, these components support *Trichoderma* survival both in soil and during storage.^61^

*Trichoderma*‐based formulations have shown promising results in terms of their survival in soils. This study demonstrated the high survival rate of *Trichoderma* in formulations based on wheat flour residues (T1). This is probably favored by the availability of carbon and nitrogen sources provided by the formulation, which favors the development of the fungus. The survival of isolate T285 is also remarkable when formulated with an inert material (T4), which could be due to different environmental factors such as pH, soil moisture, or soil temperature favoring the development of this isolate.^26^ The variability between the trial results from 2024 and 2025 may be due to soil and climatic conditions during the trials. In the 2024 trial, the re‐isolation values were higher for all treatments than in 2025, which could be due to several factors. Firstly, soil moisture and ambient temperature were higher in 2024, which favors the development of *Trichoderma* in the soil.^62^ The higher rainfall in 2025 compared to 2024 may have resulted in the bioformulates applied to the field being washed away, thus reducing their re‐isolation rate in 2025. This excess moisture, caused by the high rainfall, can also lead to reduced conidium development in the soil,^63^ thus there is no significant difference between a positive control and one of the formulates based on wheat residues.

When comparing the results obtained from the durability analysis of the *Trichoderma* bioformulations with those from the field survival trials, it becomes evident that fluctuations in environmental conditions in the field strongly influence the survival and re‐isolation of *Trichoderma*.^64^ During storage at 4 °C, *Trichoderma* remains in a dormant phase during which it requires minimal nutrients for survival.^65^, ^66^ Under these conditions, the different components of the formulation mainly serve structural and protective functions for the fungal spores.^22^ In contrast, under field conditions, *Trichoderma* must grow and develop, and therefore depends on the availability of nutrients.^26^, ^67^ Moreover, these results could be affected by other native *Trichoderma* species as well as the interesting possibilities of improving its accuracy using a real‐time PCR^68^ or a TaqMa‐qPCR probe.^69^ In this context, the ability of the residues used in the formulations to supply those nutrients becomes particularly important.^22^ Overall, the algae residue‐based bioformulation appears to be a more effective carrier, providing greater stability and long‐term preservation at low temperatures, whereas the wheat residue‐based formulation offers a higher nutrient supply capacity, promoting *Trichoderma* growth and development under field conditions.

## 5 CONCLUSION

This work has allowed us to identify a new indigenous *Trichoderma* species adapted to alkaline soils and with the ability to control the pathogens that cause black‐foot disease in vineyards. Phylogenetic analyses have allowed us to name this isolate as the new species *T. ossianense*, with high potential as a BCA in vineyards. Algae residue bioformulations of *T. ossianense* maintain spore viability at low temperatures during storage periods, whereas the wheat residue‐based formulation shows a higher ability to supply nutrients and promote *Trichoderma* development under field conditions. This study opens up a wide range of possibilities for the protection of vineyards in areas with alkaline pH soils, as well as for the prevention of black‐foot disease in nurseries and young vineyards using vegetal residue‐based formulations of *T. ossianense*.

## FUNDING INFORMATION

This research was funded by the Ministerio de Ciencia, Innovación y Universidades (Spain), which awarded a grant to Laura Zanfaño González (FPU 20/03040) and by the Research Program of the Universidad de León (2022) for the grant awarded to Daniela Ramírez Lozano. We thank Pago de Carraovejas winery for the project LOWPHWINE IDI‐20210391, “Estudio de nuevos factores relacionados con el suelo, la planta y la microbiota enológica que influyen en el equilibrio de la acidez de los vinos y en su garantía de calidad y estabilidad en climas cálidos”, which was granted by the Centro para el Desarrollo Tecnológico y la Innovacion (CDTI).

## CONFLICT OF INTEREST

The authors declare the existence of financial competing interests derived from patent numbers 516 ES2872599A1 and ES2872648A1 held by the institution (Universidad de León) as patent applicant, being four of the authors of the present manuscript part of the patents as inventors (Pedro A. Casquero, Sara Mayo‐Prieto, Guzmán Carro‐ Huerga and Álvaro Rodríguez‐González).

## Supporting information


**Figure S1.** Dual cultures of *Trichoderma ossianense* T285 (on the left of each plate) and the different pathogens of two strains of *Dactylonectria torresensis* (P112 and P140), *Dactylonectria novozelandica*, (P150), *Ilyonectria vivaria* (BV‐1924) and *Dactylonectria alcacerensis* (BV‐1240) using 8.5‐cm Petri plates. Control corresponds to the pathogen growing alone. The plates were incubated at 25 °C for 10 days.

**Figure S2.** Phylogenic tree of the genetic marker acl1 (ATP citrate lyase) using partial amino acid sequences. The sequences were retrieved from different species in paper^43^ and NCBI database (https://www.ncbi.nlm.nih.gov/).

**Figure S3.** Phylogenic tree of the genetic marker *tef1* (translation elongation factor 1‐alpha) using partial amino acid sequences. The sequences were retrieved from different species in paper.^43^

**Figure S4.** Phylogenic tree of the genetic marker *rpb2* (RNA polymerase second‐largest subunit) using partial amino acid sequences. The sequences were retrieved from different species in paper.^43^

**Table S1.** GenBank accession numbers of housekeeping genes of *Trichoderma ossianense* T285.

## Data Availability

The data that support the findings of this study are available on request from the corresponding author. The data are not publicly available due to privacy or ethical restrictions.

## References

[1] Woo SL , Ruocco M , Vinale F , Nigro M , Marra R , Lombardi N *et al*., *Trichoderma*‐based products and their widespread use in agriculture. Open Mycol J 8:71–126 (2014).

[2] Cai F and Druzhinina IS , In honor of John Bissett: authoritative guidelines on molecular identification of *Trichoderma* . Fungal Divers 107:1–69 (2021).

[3] Kubicek CP , Steindorff AS , Chenthamara K , Manganiello G , Henrissat B , Zhang J *et al*., Evolution and comparative genomics of the most common *Trichoderma* species. BMC Genomics 20:1–24 (2019). 31189469 10.1186/s12864-019-5680-7 PMC6560777

[4] Woo SL , Hermosa R , Lorito M and Monte E , *Trichoderma*: a multipurpose, plant‐beneficial microorganism for eco‐sustainable agriculture. Nat Rev Microbiol 21:312–326 (2022). 36414835 10.1038/s41579-022-00819-5

[5] Pollard‐Flamand J , Boulé J , Hart M and Úrbez‐Torres JR , Biocontrol activity of *Trichoderma* species isolated from grapevines in British Columbia against botryosphaeria dieback fungal pathogens. J Fungi 8:1–22 (2022). 10.3390/jof8040409 PMC9030288 35448640

[6] Silva‐Valderrama I , Toapanta D , Miccono M d l A , Lolas M , Díaz GA , Cantu D *et al*., Biocontrol potential of grapevine endophytic and rhizospheric fungi against trunk pathogens. Front Microbiol 11:1–13 (2021). 10.3389/fmicb.2020.614620 PMC7817659 33488557

[7] Mutawila C , Halleen F and Mostert L , Optimisation of time of application of Trichoderma biocontrol agents for protection of grapevine pruning wounds. Aust J Grape Wine Res 22:279–287 (2016).

[8] Úrbez‐Torres JR , Tomaselli E , Pollard‐Flamand J , BoulÉ J , Gerin D and Pollastro S , Characterization of *Trichoderma* isolates from southern Italy, and their potential biocontrol activity against grapevine trunk disease fungi. Phytopathol Mediterr 59:425–439 (2020).

[9] Langa‐Lomba N , Martín‐Ramos P , Casanova‐Gascón J , Julián‐Lagunas C and González‐García V , Potential of native *Trichoderma* strains as antagonists for the control of fungal wood pathologies in young grapevine plants. Agronomy 12:1–15 (2022).

[10] Mondello V , Songy A , Battiston E , Pinto C , Coppin C , Trotel‐Aziz P , *et al*. Grapevine trunk diseases: a review of fifteen years of trials for their control with chemicals and biocontrol agents., Plant Dis 102:1189–1217 (2018). 30673583 10.1094/PDIS-08-17-1181-FE

[11] Carro‐Huerga G , Mayo‐Prieto S , Rodríguez‐González Á , Cardoza RE , Gutiérrez S and Casquero PA , Vineyard management and physicochemical parameters of soil affect native *Trichoderma* populations, sources of biocontrol agents against *Phaeoacremonium minimum* . Plants 12:1–20 (2023). 10.3390/plants12040887 PMC9966749 36840235

[12] Pedrero‐Méndez A , Insuasti HC , Neagu T , Illescas M , Rubio MB , Monte E *et al*., Why is the correct selection of *Trichoderma* strains important? The case of wheat endophytic strains of *T. harzianum* and *T. simmonsii* . J Fungi 7:1–21 (2021). 10.3390/jof7121087 PMC8704890 34947069

[13] Agustí‐Brisach C and Armengol J , Black‐foot disease of grapevine: an update on taxonomy, epidemiology and management strategies. Phytopathol Mediterr 52:245–261 (2013).

[14] Berlanas C , Ojeda S , López‐Manzanares B , Andrés‐Sodupe M , Bujanda R , del Pilar Martínez‐Diz M *et al*., Occurrence and diversity of black‐foot disease fungi in symptomless grapevine nursery stock in Spain. Plant Dis 104:94–104 (2020). 31738690 10.1094/PDIS-03-19-0484-RE

[15] Halleen F and Fourie PH , A review of black foot disease of grapevine. Phytopathol Mediterr 45:55–67 (2006).

[16] Gramaje D , Úrbez‐Torres JR and Sosnowski MR , Managing grapevine trunk diseases with respect to etiology and epidemiology: current strategies and future prospects. Plant Dis 102:12–39 (2018). 30673457 10.1094/PDIS-04-17-0512-FE

[17] Leal C , Gramaje D , Fontaine F , Richet N , Trotel‐Aziz P and Armengol J , Evaluation of *Bacillus subtilis* PTA‐271 and *Trichoderma atroviride* SC1 to control Botryosphaeria dieback and black‐foot pathogens in grapevine propagation material. Pest Manag Sci 79:1674–1683 (2023). 36573682 10.1002/ps.7339

[18] Berbegal M , Ramón‐Albalat A , León M and Armengol J , Evaluation of long‐term protection from nursery to vineyard provided by *Trichoderma atroviride* SC1 against fungal grapevine trunk pathogens. Pest Manag Sci 76:967–977 (2019). 31472038 10.1002/ps.5605

[19] Fierer N , Embracing the unknown: disentangling the complexities of the soil microbiome. Nat Rev Microbiol 15:579–590 (2017). 28824177 10.1038/nrmicro.2017.87

[20] Zarraonaindia I , Owens SM , Weisenhorn P , West K , Hampton‐Marcell J , Lax S *et al*., The soil microbiome influences grapevine‐associated microbiota. MBio 6:1–10 (2015). 10.1128/mBio.02527-14 PMC4453523 25805735

[21] van Jaarsveld WJ , Halleen F , Bester MC , Pierron RJG , Stempien E and Mostert L , Investigation of *Trichoderma* species colonization of nursery grapevines for improved management of black foot disease. Pest Manag Sci 77:397–405 (2021). 32741056 10.1002/ps.6030

[22] Martínez Y , Ribera J , Schwarze FWMR and De France K , Biotechnological development of *Trichoderma*‐based formulations for biological control. Appl Microbiol Biotechnol 107:5595–5612 (2023). 37477696 10.1007/s00253-023-12687-x PMC10439859

[23] Preininger C , Sauer U , Bejarano A and Berninger T , Concepts and applications of foliar spray for microbial inoculants. Appl Microbiol Biotechnol 102:7265–7282 (2018). 29961100 10.1007/s00253-018-9173-4

[24] Vassilev N , Vassileva M , Martos V , del Garcia Moral LF , Kowalska J , Tylkowski B *et al*., Formulation of microbial inoculants by encapsulation in natural polysaccharides: focus on beneficial properties of carrier additives and derivatives. Front Plant Sci 11:523157 (2020). 10.3389/fpls.2020.00270 PMC7077505 32211014

[25] Khan S , Bagwan NB , Iqbal MA and Tamboli RR , Mass multiplication and shelf life of liquid fermented final product of *Trichoderma viride* in different formulations. Adv Biores 2:178–182 (2011).

[26] Longa CMO , Pertot I and Tosi S , Ecophysiological requirements and survival of a *Trichoderma atroviride* isolate with biocontrol potential. J Basic Microbiol 48:269–277 (2008). 18720503 10.1002/jobm.200700396

[27] Khan A , Singh AV , Gautam SS , Agarwal A , Punetha A , Upadhayay VK *et al*., Microbial bioformulation: a microbial assisted biostimulating fertilization technique for sustainable agriculture. Front Plant Sci 14:1–22 (2023). 10.3389/fpls.2023.1270039 PMC10749938 38148858

[28] Shokrkar H and Zamani M , Comparison of microalgae and other common nitrogen sources for cellulase production. Biomass Convers Biorefin 14:24049–24059 (2023).

[29] Ammar EE , Aioub AAA , Elesawy AE , Karkour AM , Mouhamed MS , Amer AA *et al*., Algae as bio‐fertilizers: between current situation and future prospective. Saudi J Biol Sci 29:3083–3096 (2022). 35360501 10.1016/j.sjbs.2022.03.020 PMC8961072

[30] Luskar L , Polanšek J , Hladnik A and Čeh B , On‐farm composting of hop plant green waste—chemical and biological value of compost. Appl Sci (Switz) 12:1–15 (2022).

[31] Casquero PA , Mayo‐Prieto S , Rodríguez‐González Á , Carro‐Huerga G , Álvarez García S , Porteus Álvarez AJ *et al*., Recubrimiento de semilla que comprende un agente de biocontrol y conos de lúpulo (España, No ES2872599A1) (2021).

[32] Mayo‐Prieto S , Porteous‐Álvarez AJ , Carro‐Huerga G , Zanfaño L , Ramírez‐Lozano D *et al*., Hop waste seed coating (pilling) as circular bioeconomic alternative to improve seed germination and *Trichoderma* development. Agri 15:1–19 (2025).

[33] Lawrence DP , Nouri MT and Trouillas FP , Taxonomy and multi‐locus phylogeny of cylindrocarpon‐like species associated with diseased roots of grapevine and other fruit and nut crops in California. Fungal Syst Evol 4:59–75 (2019). 32467907 10.3114/fuse.2019.04.06 PMC7241681

[34] Zanfaño L , Carro‐Huerga G , Rodríguez‐González Á , Mayo‐Prieto S , Cardoza RE , Gutiérrez S *et al*., *Trichoderma carraovejensis*: a new species from vineyard ecosystem with biocontrol abilities against grapevine trunk disease pathogens and ecological adaptation. Front Plant Sci 15:1–15 (2024). 10.3389/fpls.2024.1388841 PMC11148300 38835860

[35] Gutiérrez S , McCormick SP , Cardoza RE , Kim H‐S , Yugueros LL , Vaughan MM *et al*., Distribution, function, and evolution of a gene essential for trichothecene toxin biosynthesis in *Trichoderma* . Front Microbiol 12:1–45 (2021). 10.3389/fmicb.2021.791641 PMC8675399 34925301

[36] Kumar S , Stecher G , Li M , Knyaz C and Tamura K , MEGA X: molecular evolutionary genetics analysis across computing platforms. Mol Biol Evol 35:1547–1549 (2018). 29722887 10.1093/molbev/msy096 PMC5967553

[37] Vaidya G , Lohman DJ and Meier R , SequenceMatrix: concatenation software for the fast assembly of multi‐gene datasets with character set and codon information. Cladistics 27:171–180 (2011). 34875773 10.1111/j.1096-0031.2010.00329.x

[38] Nguyen L‐T , Schmidt HA , von Haeseler A and Minh BQ , IQ‐TREE: a fast and effective stochastic algorithm for estimating maximum‐likelihood phylogenies. Mol Biol Evol 32:268–274 (2015). 25371430 10.1093/molbev/msu300 PMC4271533

[39] Minh BQ , Hahn MW and Lanfear R , New methods to calculate concordance factors for phylogenomic datasets. Mol Biol Evol 37:2727–2733 (2020). 32365179 10.1093/molbev/msaa106 PMC7475031

[40] Minh BQ , Schmidt HA , Chernomor O , Schrempf D , Woodhams MD , Von Haeseler A *et al*., IQ‐TREE 2: new models and efficient methods for phylogenetic inference in the genomic era. Mol Biol Evol 37:1530–1534 (2020). 32011700 10.1093/molbev/msaa015 PMC7182206

[41] Jaklitsch WM and Voglmayr H , Biodiversity of *Trichoderma (Hypocreaceae)* in southern Europe and Macaronesia. Stud Mycol 80:1–87 (2015). 26955191 10.1016/j.simyco.2014.11.001 PMC4779795

[42] Prasad RD , Poorna Chandrika KS V , Desai S , Greeshma K and Vijaykumar S , Development, Production, and Storage of Trichoderma Formulations for Agricultural Applications. Springer, Cham, pp. 371–385 (2022).

[43] Kumar R , Rajan C and Author C , Shelf life studies on solid and liquid formulations of *Trichoderma harzianum* and *Pseudomonas fluorescens* . Pharma Innov J 11:866–873 (2022).

[44] Gotor‐Vila A , Usall J , Torres R , Solsona C and Teixidó N , Enhanced shelf‐life of the formulated biocontrol agent *Bacillus amyloliquefaciens* CPA‐8 combining diverse packaging strategies and storage conditions. Int J Food Microbiol 290:205–213 (2019). 30366262 10.1016/j.ijfoodmicro.2018.10.013

[45] Harman GE and Kubicek CP , Trichoderma and Gliocladium, in Basic Biology, Taxonomy and Genetics, Vol. 1. CRC Press, London (1998).

[46] Zhao R , Chen KY , Mao LJ and Zhang CL , Eleven new species of *Trichoderma* (Hypocreaceae, Hypocreales) from China. Mycology 16:1–31 (2024). 40083403 10.1080/21501203.2024.2330400 PMC11899217

[47] Samuels GJ , Dodd S , Lu BS , Petrini O , Schroers HJ and Druzhinina IS , The *Trichoderma koningii* aggregate species. Stud Mycol 56:67–133 (2006). 18490990 10.3114/sim.2006.56.03 PMC2104733

[48] Griffiths RI , Thomson BC , James P , Bell T , Bailey M and Whiteley AS , The bacterial biogeography of British soils. Environ Microbiol 13:1642–1654 (2011). 21507180 10.1111/j.1462-2920.2011.02480.x

[49] Benítez T , Rincón AM , Limón MC and Codón AC , Biocontrol mechanism of *Trichoderma* strains. Int Microbiol 7:249–260 (2004). 15666245

[50] Kredics L , Antal Z , Manczinger L , Szekeres A , Kevei F and Nagy E , Influence of environmental parameters on *Trichoderma* strains with biocontrol potential, trichoderma strains with biocontrol potential. Food Technol Biotechnol 41:37–42 (2003).

[51] Pan X , Zhang S , Zhong Q , Gong G , Wang G , Guo X *et al*., Effects of soil chemical properties and fractions of Pb, Cd, and Zn on bacterial and fungal communities. Sci Total Environ 715:1–10 (2020). 10.1016/j.scitotenv.2020.136904 32007886

[52] Türkkan M , Evaluation of inhibitory effect of organic and inorganic salts against *Ilyonectria liriodendri*, the causal agent of root rot disease of kiwifruit. J Phytopathol 163:567–577 (2015).

[53] Reis P , Cabral A , Nascimento T , Oliveira H and Rego C , Diversity of *Ilyonectria* species in a young vineyard affected by black foot disease. Phytopathol Mediterr 52:335–346 (2013).

[54] Van Jaarsveld WJ , Halleen F and Mostert L , In vitro screening of *Trichoderma* isolates for biocontrol of black foot disease pathogens. Phytopathol Mediterr 59:465–471 (2020).

[55] Pereira MMA , Moraes LC , Mogollón MCT , Borja CJF , Duarte M , Buttrós VHT *et al*., Cultivating biodiversity to harvest sustainability: vermicomposting and inoculation of microorganisms for soil preservation and resilience. Agronomy 13:1–26 (2023).

[56] Martínez Y , Heeb M , Kalač T , Gholam Z , Schwarze FWMR , Nyström G , *et al*. Biopolymer‐based emulsions for the stabilization of *Trichoderma atrobrunneum* conidia for biological control, Appl Microbiol Biotechnol 107:1465–1476 (2023). 36683057 10.1007/s00253-023-12381-y PMC9898383

[57] Casquero PA , Mayo‐Prieto S , Rodríguez‐González Á , Carro‐Huerga G , Älvarez García S , Porteus Álvarez AJ *et al*., Recubrimiento de semilla que contiene un agente de biocontrol y *Sargassum muticum* (España, No ES2872648A1) (2021).

[58] Cobos R , Mateos RM , Álvarez‐Pérez JM , Olego MA , Sevillano S , González‐García S , *et al*. Effectiveness of natural antifungal compounds in controlling infection by grapevine trunk disease pathogens through pruning wounds, Appl Environ Microbiol 81:6474–6483 (2015). 26162882 10.1128/AEM.01818-15 PMC4542253

[59] Kinay P and Yildiz M , The shelf life and effectiveness of granular formulations of *Metschnikowia pulcherrima* and *Pichia guilliermondii* yeast isolates that control postharvest decay of citrus fruit. Biological Control 45:433–440 (2008).

[60] Bhattacharyya SK and Basu MK , Kaolin powder as a fungal carrier. Appl Environ Microbiol 44:751–753 (1982). 16346103 10.1128/aem.44.3.751-753.1982 PMC242087

[61] Chammem H , Antonielli L , Nesler A , Pindo M and Pertot I , Effect of a wood‐based carrier of *Trichoderma atroviride* SC1 on the microorganisms of the soil. J Fungi 7:1–18 (2021). 10.3390/jof7090751 PMC8467423 34575789

[62] Eastburn DM and Butler EE , Effects of soil moisture and temperature on the saprophytic ability of *Trichoderma harzianum* . Mycologia 83:257–263 (1991).

[63] Cao Q , Liang Y , Tian Y , Lian H , Jiang X and Li M , Survival dynamics of *Trichoderma longibrachiatum* Tr58 in conidia‐ and chlamydospore‐amended soils with different moisture levels. Agriculture (Switzerland) 13:1–10 (2023).

[64] Carro‐Huerga G , Mayo‐Prieto S , Rodríguez‐González Á , Álvarez‐García S , Gutiérrez S and Casquero PA , The influence of temperature on the growth, sporulation, colonization, and survival of *Trichoderma* spp. in grapevine pruning wounds. Agronomy 11:1–18 (2021).

[65] Swaminathan J , van Koten C , Henderson HV , Jackson TA and Wilson MJ , Formulations for delivering *Trichoderma atroviridae* spores as seed coatings, effects of temperature and relative humidity on storage stability. J Appl Microbiol 120:425–431 (2016). 26600429 10.1111/jam.13006

[66] Cortés‐Rojas D , Santos‐Diaz A , Torres‐Torres L , Zapata‐Narváez Y , Beltrán‐Acosta C and Cruz‐Barrera M , *Trichoderma koningiopsis* survival on coated seeds and effect on plant growth promotion in rice (*Oryza sativa*). Curr Microbiol 80:1–11 (2022). 36460904 10.1007/s00284-022-03076-0

[67] Šimkovič M , Olejníková P , Mat'at'a M , Žemla P , Vilimová V , Farkašová L *et al*., Nutrient transport into germinating *Trichoderma atroviride* conidia and development of its driving force. Microbiology (United Kingdom) 161:1240–1250 (2015). 10.1099/mic.0.000079 25777081

[68] Savazzini F , Longa CMO , Pertot I and Gessler C , Real‐time PCR for detection and quantification of the biocontrol agent *Trichoderma atroviride* strain SC1 in soil. J Microbiol Methods 73:185–194 (2008). 18375004 10.1016/j.mimet.2008.02.004

[69] Gerin D , Pollastro S , Raguseo C , Angelini RMDM and Faretra F , A ready‐to‐use single‐ and duplex‐TaqMan‐qPCR assay to detect and quantify the biocontrol agents *Trichoderma asperellum* and *Trichoderma gamsii* . Front Microbiol 9:1–9 (2018). 30233545 10.3389/fmicb.2018.02073 PMC6127317

