# Supplementary material for: Vegetal residue‐based formulation of Trichoderma ossianense, a new indigenous vineyard species adapted to alkaline pH with potential biocontrol ability against Black‐foot disease pathogens
Source: Pest Manag Sci. 2025 Dec 6;82(4):2910–24. doi: 10.1002/ps.70417 (PMC12976189; doi:10.1002/ps.70417)
Supplement: Supplementary file 1 — Figure S1. Dual cultures of Trichoderma ossianense T285 (on the left of each plate) and the different pathogens of two strains of Dactylonectria torresensis (P112 and P140), Dactylonectria novozelandica, (P150), Ilyonectria vivaria (BV‐1924) and Dactylonectria alcacerensis (BV‐1240) using 8.5‐cm Petri plates. Control corresponds to the pathogen growing alone. The plates were incubated at 25 °C for 10 days. [file PS-82-2910-s003.docx]

Supplementary Material
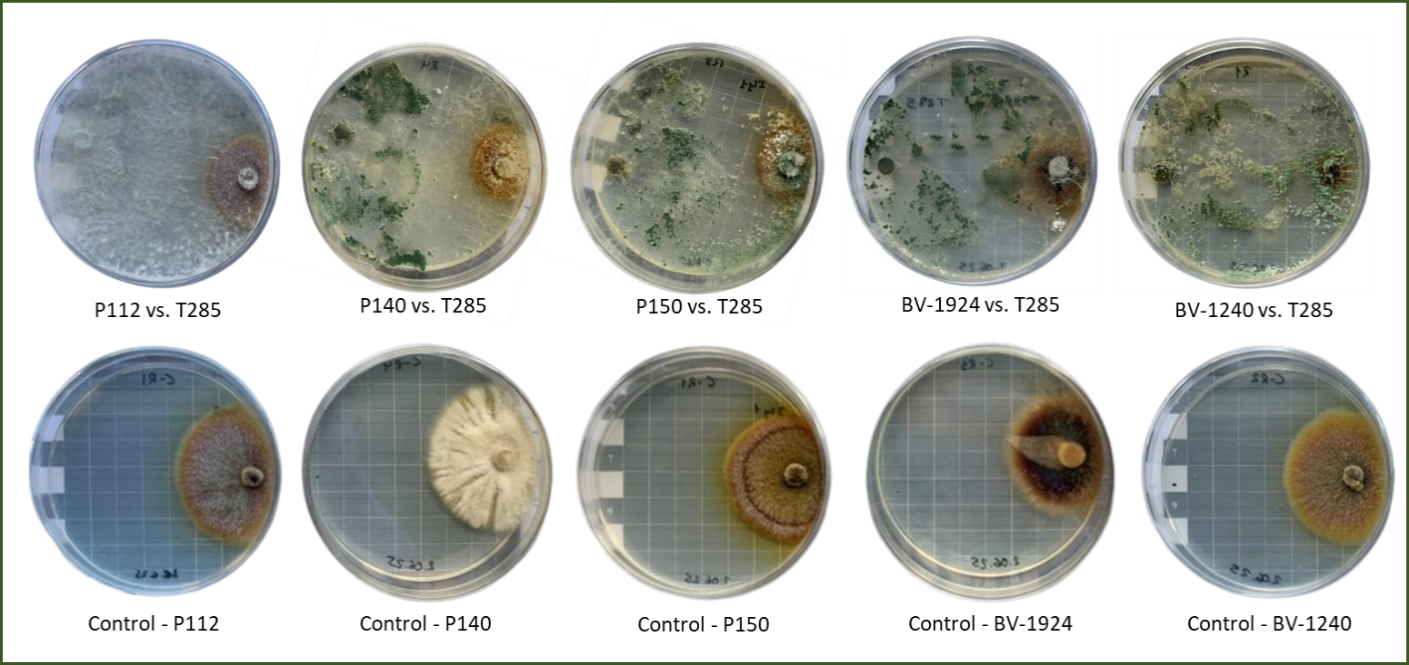


**Supplementary Figure S1.** Dual cultures of Trichoderma ossianense T285 (on the left of each plate) and the different pathogens two strains of Dactylonectria torresensis (P112 and P140), Dactylonectria novozelandica, (P150), Ilyonectria vivaria (BV-1924) and Dactylonectria alcacerensis (BV-1240). Using 8.5 cm Petri plates. Control corresponds to the pathogen growing alone. The plates were incubated at 25 ºC for ten days.
