# Supplementary material for: Vegetal residue‐based formulation of Trichoderma ossianense, a new indigenous vineyard species adapted to alkaline pH with potential biocontrol ability against Black‐foot disease pathogens
Source: Pest Manag Sci. 2025 Dec 6;82(4):2910–24. doi: 10.1002/ps.70417 (PMC12976189; doi:10.1002/ps.70417)
Supplement: Supplementary file 2 — Figure S2. Phylogenic tree of the genetic marker acl1 (ATP citrate lyase) using partial amino acid sequences. The sequences were retrieved from different species in paper 43 and NCBI database (https://www.ncbi.nlm.nih.gov/). [file PS-82-2910-s002.docx]

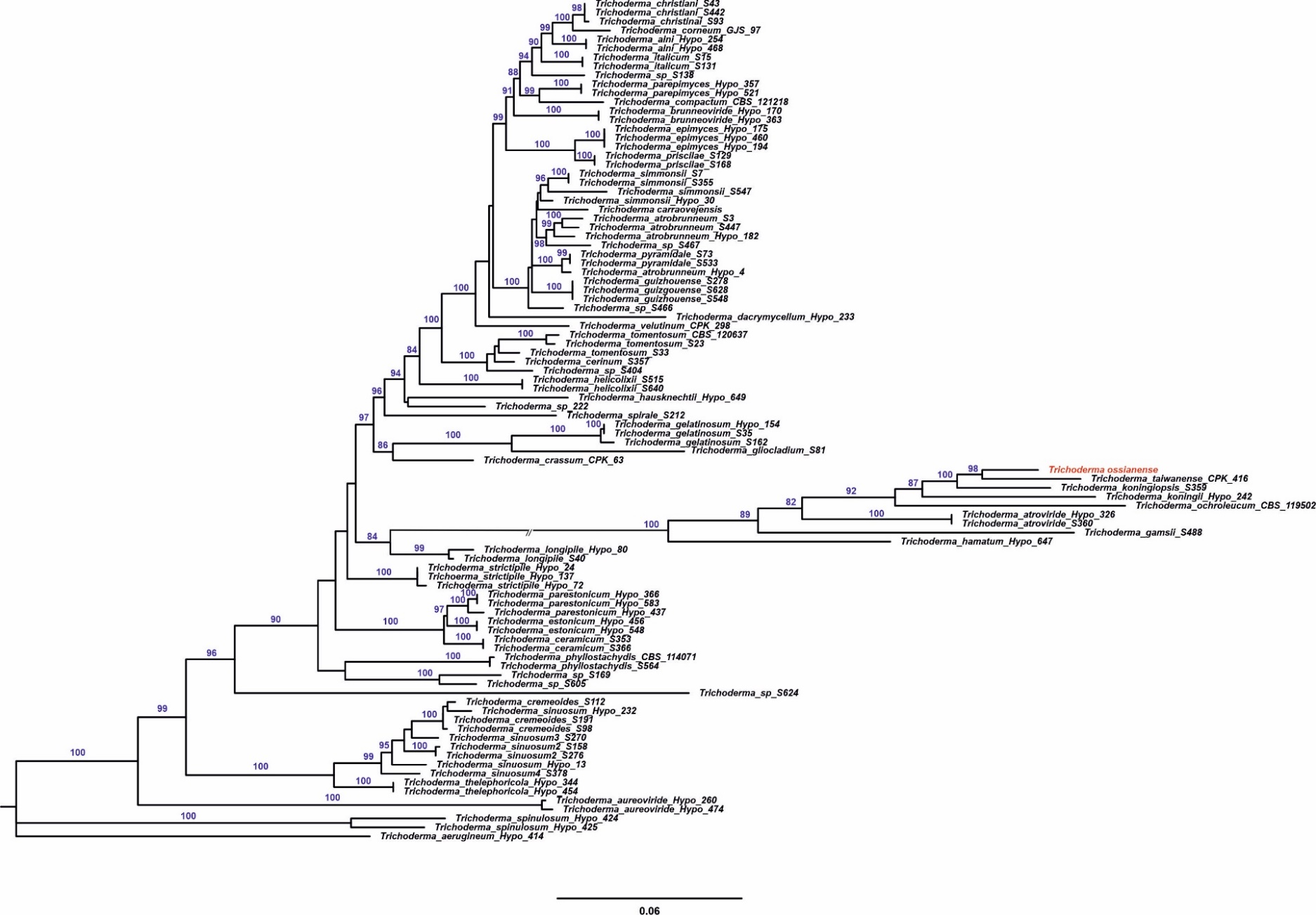


**Supplementary Figure S2**. Phylogenic tree of the genetic marker acl1 (ATP citrate lyase) using partial amino acid sequences. The sequences were retrieved from different species in paper^43^ and NCBI database (<https://www.ncbi.nlm.nih.gov/>).
