# Supplementary material for: Vegetal residue‐based formulation of Trichoderma ossianense, a new indigenous vineyard species adapted to alkaline pH with potential biocontrol ability against Black‐foot disease pathogens
Source: Pest Manag Sci. 2025 Dec 6;82(4):2910–24. doi: 10.1002/ps.70417 (PMC12976189; doi:10.1002/ps.70417)
Supplement: Supplementary file 3 — Figure S3. Phylogenic tree of the genetic marker tef1 (translation elongation factor 1‐alpha) using partial amino acid sequences. The sequences were retrieved from different species in paper. 43 [file PS-82-2910-s005.docx]

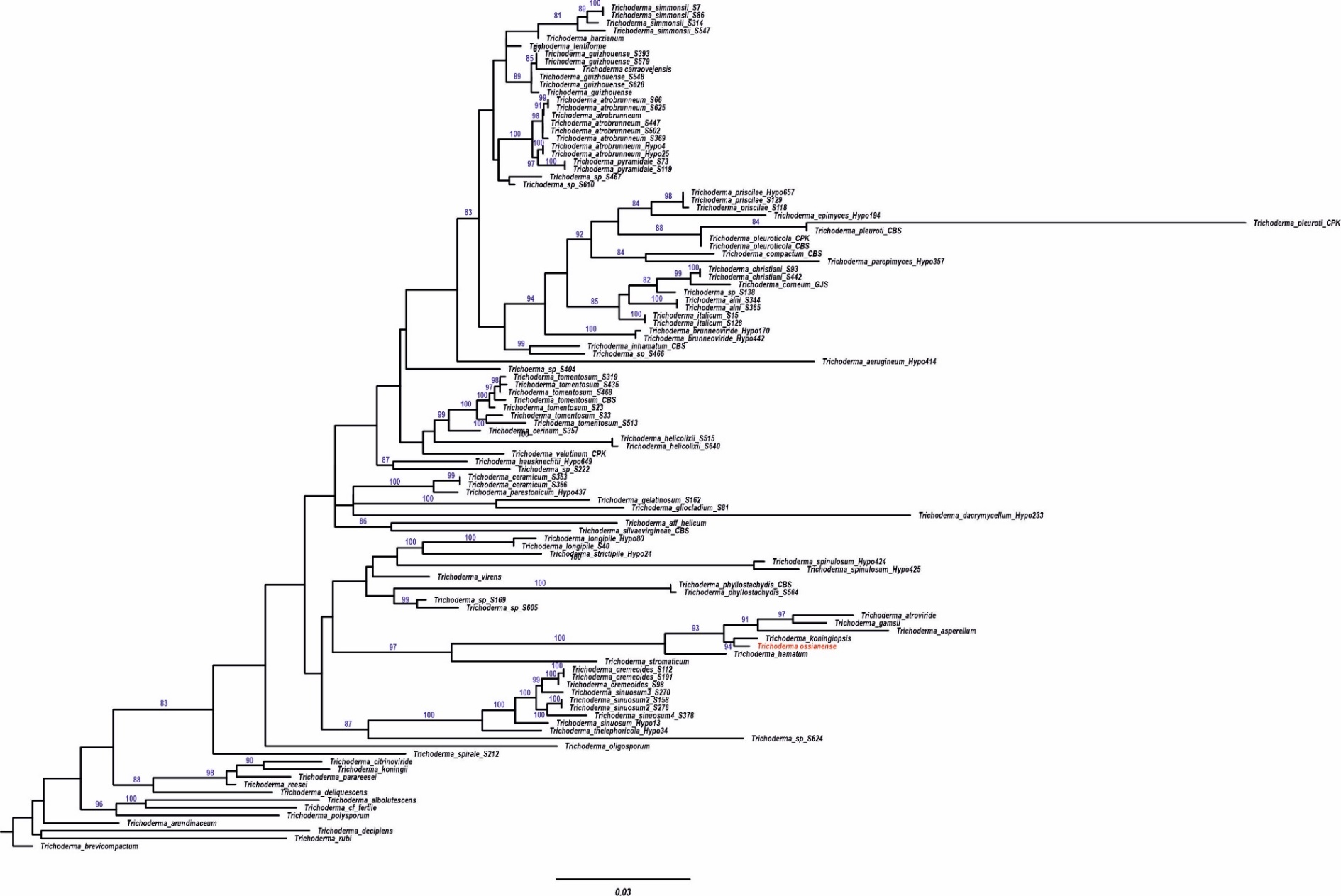


**Supplementary Figure S3**. Phylogenic tree of the genetic marker tef1 (translation elongation factor 1-alpha) using partial amino acid sequences. The sequences were retrieved from different species in paper.^43^
