# Supplementary material for: Vegetal residue‐based formulation of Trichoderma ossianense, a new indigenous vineyard species adapted to alkaline pH with potential biocontrol ability against Black‐foot disease pathogens
Source: Pest Manag Sci. 2025 Dec 6;82(4):2910–24. doi: 10.1002/ps.70417 (PMC12976189; doi:10.1002/ps.70417)
Supplement: Supplementary file 4 — Figure S4. Phylogenic tree of the genetic marker rpb2 (RNA polymerase second‐largest subunit) using partial amino acid sequences. The sequences were retrieved from different species in paper. 43 [file PS-82-2910-s004.docx]

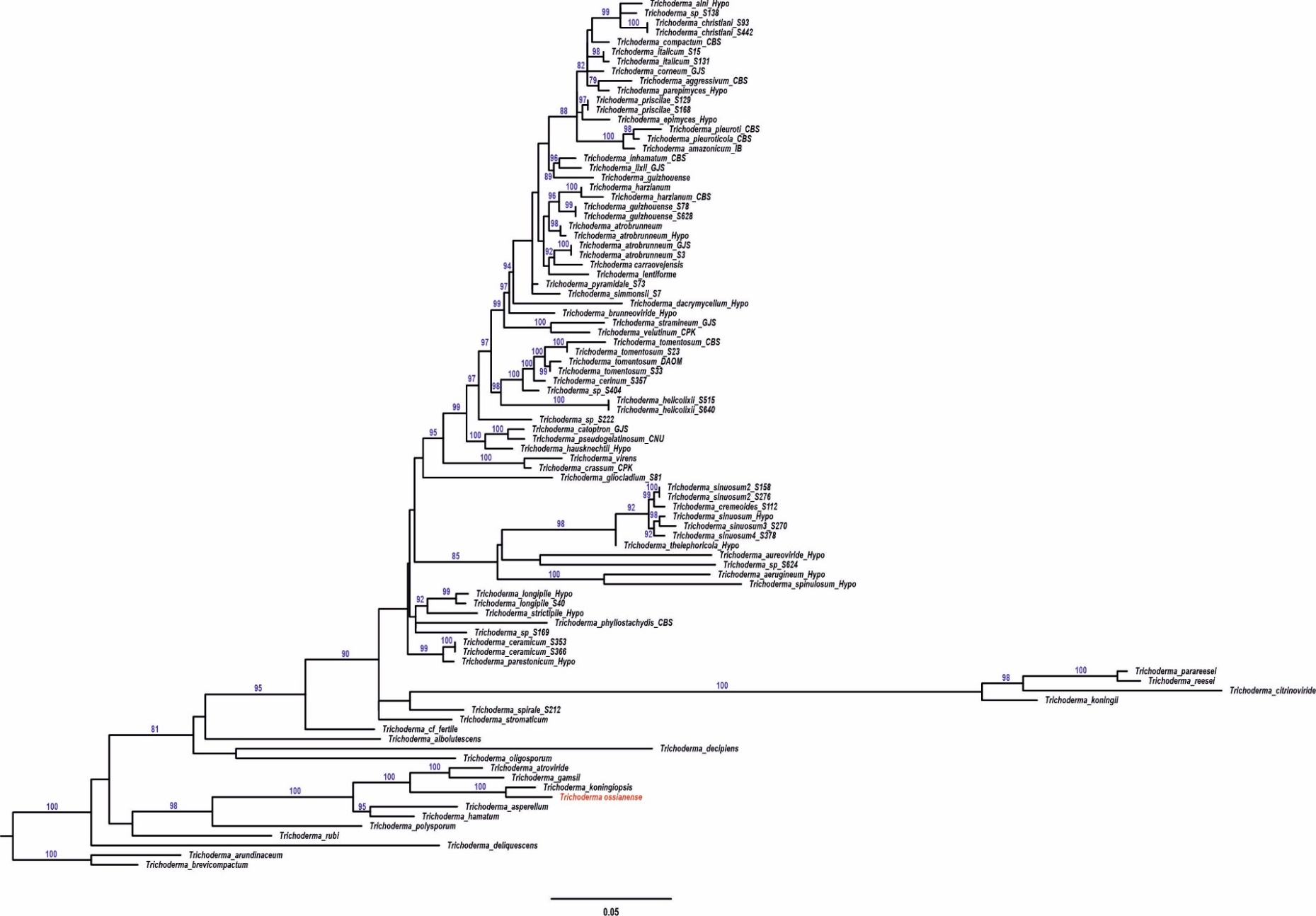


**Supplementary Figure S4**. Phylogenic tree of the genetic marker rpb2 (RNA polymerase 2nd largest subunit) using partial amino acid sequences. The sequences were retrieved from different species in paper.^43^
