# Supplementary material for: Vegetal residue‐based formulation of Trichoderma ossianense, a new indigenous vineyard species adapted to alkaline pH with potential biocontrol ability against Black‐foot disease pathogens
Source: Pest Manag Sci. 2025 Dec 6;82(4):2910–24. doi: 10.1002/ps.70417 (PMC12976189; doi:10.1002/ps.70417)
Supplement: Supplementary file 5 — Table S1. GenBank accession numbers of housekeeping genes of Trichoderma ossianense T285. [file PS-82-2910-s001.docx]

Supplementary Material

**Supplementary Table S1.** GenBank accession numbers of housekeeping genes of Trichoderma ossianense T285

| **Gene** | **Predicted gene function** | **Accession number** |
| --- | --- | --- |
| *cpr1* | Cytochrome P450 reductase | PV056193 |
| *dpa1* | DNA polymerase alpha subunit | PV056177 |
| *dpd1* | DNA polymerase delta subunit | PV056178 |
| *erg1* | ergosterol monooxygenase/oxidase | PV056179 |
| *fas1* | Fatty acid synthase alpha subunit | PV056180 |
| *fas2* | Fatty acid synthase beta subunit | PV056181 |
| *lcb1* | Sphinganine palmitoyl transferase subunit 1 | PV056182 |
| *mcm7* | DNA replication licensing factor | PV056183 |
| *pgk1* | Phosphoglycerate kinase | PV056184 |
| *rpb1* | RNA polymerase largest subunit | PV056185 |
| *sph1* | Sphinganine N acyl transferase subunit 1 | PV056186 |
| *top1* | Topoisomerase | PV056187 |
| *tps1* | Trehalose phosphate synthase | PV056188 |
| *tsr1* | Ribosomal biogenesis protein | PV056189 |
| *tub1* | Tubulin alpha subunit | PV056190 |
| *tub2* | Beta-tubulin | PV056191 |
| *ubt1* | Ubiquitin thiolesterase | PV056192 |
